# PRMT5-mediated homologous recombination repair is essential to maintain genomic integrity of neural progenitor cells

**DOI:** 10.1007/s00018-024-05154-x

**Published:** 2024-03-08

**Authors:** Ya-Jun Wang, Jian-Bo Cao, Jing Yang, Tong Liu, Hua-Li Yu, Zi-Xuan He, Shi-Lai Bao, Xiao-Xiao He, Xiao-Juan Zhu

**Affiliations:** 1 Key Laboratory of Molecular Epigenetics, Ministry of Education, Institute of Genetics and Cytology, Northeast Normal University, Changchun, 130024 China; 2 State Key Laboratory of Molecular and Developmental Biology, Institute of Genetics and Developmental Biology, Chinese Academy of Sciences, Beijing, 100101 China

**Keywords:** Genomic integrity, Microcephaly, Apoptosis, DNA double-strand break, DNA repair, Histone arginine methylation

## Abstract

Maintaining genomic stability is a prerequisite for proliferating NPCs to ensure genetic fidelity. Though histone arginine methylation has been shown to play important roles in safeguarding genomic stability, the underlying mechanism during brain development is not fully understood. Protein arginine N-methyltransferase 5 (PRMT5) is a type II protein arginine methyltransferase that plays a role in transcriptional regulation. Here, we identify PRMT5 as a key regulator of DNA repair in response to double-strand breaks (DSBs) during NPC proliferation. *Prmt5*^*F/F*^*; Emx1-Cre* (cKO-Emx1) mice show a distinctive microcephaly phenotype, with partial loss of the dorsal medial cerebral cortex and complete loss of the corpus callosum and hippocampus. This phenotype is resulted from DSBs accumulation in the medial dorsal cortex followed by cell apoptosis. Both RNA sequencing and in vitro DNA repair analyses reveal that PRMT5 is required for DNA homologous recombination (HR) repair. PRMT5 specifically catalyzes H3R2me2s in proliferating NPCs in the developing mouse brain to enhance HR-related gene expression during DNA repair. Finally, overexpression of BRCA1 significantly rescues DSBs accumulation and cell apoptosis in PRMT5-deficient NSCs. Taken together, our results show that PRMT5 maintains genomic stability by regulating histone arginine methylation in proliferating NPCs.

**Supplementary Information:**

The online version contains supplementary material available at 10.1007/s00018-024-05154-x.

## Introduction

During mammalian brain development, accurate DNA replication is essential for the delivery of genetic information in neural progenitor cells (NPCs) [1]. Due to the high DNA replication rate, DNA damage, especially DNA double-strand breaks (DSBs), inevitably arises in NPCs [2]. Appropriate responses to DNA damage are required to safeguard genomic integrity [3–5]. NPCs normally employ two kinds of repair pathways to maintain genomic integrity, which are homologous recombination (HR) and nonhomologous end joining (NHEJ) [6–8]. HR uses homologous sequences to faithfully repair DSBs during the S and G2 phases of the cell cycle with low efficiency and high fidelity, whereas NHEJ ligates free DNA ends throughout the cell cycle in a homology-independent manner that tends to introduce indels or structural rearrangements [6]. During DNA damage response (DDR) and DNA repair process, post-translational modifications of histones are important for the transcription and recruitment of DNA repair proteins. Numerous studies have confirmed that arginine methylation plays critical roles in DNA damage response [9].

Protein arginine methyltransferase 5 (PRMT5) is a type II arginine methyltransferase [10] that catalyzes symmetric dimethylation of histone (H) to generate H2A arginine 3 (H2AR3me2s), H3 arginine 2 (H3R2me2s), H3 arginine 8 (H3R8me2s) and H4 arginine 3 (H4R3me2s) to take part in many important cellular processes, including cell proliferation, differentiation, and cell cycle progression [11–14]. Typically, PRMT5 acts as a gene repressor by mediating symmetric dimethylation of H3R8 and H4R3, which are markers of gene transcription inhibition. PRMT5 also enhances transcription through symmetric dimethylation of H3R2 preventing recruitment of repressor complexes [15]. Recently, accumulating evidence has indicated that PRMT5 is involved in the DNA damage response in a variety of cells [16–20]. PRMT5 has been shown to promote homologous recombination (HR) in a TIP60-related manner in cancer cells and hematopoietic stem/progenitor cells (HSPCs) [17, 18]. In addition, PRMT5 deficiency alters the methylation status of E2F1 and attenuates DNA repair in myeloproliferative neoplasms (MPNs) [20].

Global PRMT5 knockout (KO) mice die early during embryonic development [11], making it impossible to study the role of PRMT5 in the developing brain. *Nestin-Cre*-mediated conditional deletion of PRMT5 (cKO-Nes) affects the overall brain development [21]. In vitro analysis has shown that PRMT5 deficiency significantly reduces NPC proliferation, but this defect is not observed in vivo. RNA-sequence analysis suggests that activation of the p53 pathway in the PRMT5 deletion brain leads to cell apoptosis. However, p53 deletion only partially rescues the size decrease in the PRMT5 deletion brain. Thus, the role of PRMT5 in maintaining NPC proliferation and differentiation is still unclear.

Here, we used conditional gene knockout via Cre/LoxP technology to determine the role of PRMT5 during brain development by using *Emx1-Cre* to conditionally knockout *Prmt5* in dorsal cortical NPCs. These mice showed severe anatomical abnormalities of the neocortex, including size reduction and complete loss of the hippocampus and corpus callosum. PRMT5 deletion in NPCs, but not in differentiated neurons, led to DSBs accumulation and cell apoptosis. RNA sequencing and in vitro analysis both revealed that PRMT5 deficiency selectively impaired HR repair. PRMT5-catalyzed H3R2me2s activated HR-related gene expression during DNA damage response in proliferating NPCs. Overexpression of BRCA1 rescued DSBs accumulation and cell apoptosis in PRMT5-deficient NSCs. Taken together, our results revealed a cell type specific function of PRMT5 in regulating histone arginine methylation to maintain genetic stability in developing NPCs.

## Materials and methods

### Animals

All animals used in the study were maintained and operated in strict compliance with the requirements of the Institutional Animal Care and Use Committee of the Northeast Normal University, China (NENU/IACUC, AP20171008). Mice were housed under sterile conditions with a 12-h light/12-h dark cycle. Females were bred overnight with males. Noon on the day after breeding was considered E0.5, and the day of birth was considered postnatal Day 0 (P0). The backgrounds of all the mice were C57BL/6.

The *Prmt5*^*F/F*^ mice were kindly provided by Prof. Shilai Bao (Institute of Genetics and Developmental Biology, CAS, China). *Emx1-Cre* mice (Jax No. 005628), *Nex-Cre* mice (Goebbels et al. 2006) and Ai9D mice were initially obtained from The Jackson Laboratory and were maintained in the C57BL/6 background. *Trp53*^*−/−*^ mice were purchased from BIOCYTOGEN company (Beijing, China).

### Antibodies

Primary antibodies used in this study included rabbit anti-PRMT5 (Abcam, ab109451), rabbit anti-CDP (Santa Cruz, sc-101003), rat anti-Ctip2 (Abcam, ab18465), rabbit anti-Foxp2 (Abcam, ab16046), rabbit anti-TBR2 / Eomes antibody (Abcam, AB23345), rabbit anti-Pax6 (COVANCE, PRB-278P), rabbit anti-Ki67 (Abcam, ab66155), rat anti-BrdU (Abcam, ab6326), rabbit anti-CC3 (CST, 9661), rabbit anti-γH2AX (CST, 9718), rabbit anti-BRCA1 (ABclonal, A11034), rabbit anti-RAD51 (ABclonal, A6268), rabbit anti-53BP1 (ABclonal, A5757), rabbit anti-KU70 (ABclonal, A0883), rabbit anti-ATM (ABclonal, A18257), rabbit anti-p-ATM (ABclonal, AP0008), rabbit anti-p53 (ABclonal, A3185), rabbit anti-p-p53 (ABclonal, AP0083), mouse anti-FLAG (Sigma‒Aldrich, F7425), rabbit anti-H4R3me2s (ABclonal, A3159), rabbit anti-H3R2me2s (ABclonal, A2373), and rabbit anti-H3R8me2s (ABclonal, A2374).

The secondary antibodies used in this study included anti-mouse IgG-HRP (Santa Cruz, sc-2005), donkey anti-mouse Alexa Fluor 488 (Invitrogen, A21202), goat anti-rabbit IgG-HRP (Invitrogen, PI31460), donkey anti-rabbit Alexa Fluor 546 (Invitrogen, A10040), and donkey anti-mouse Alexa Fluor 546 (Invitrogen, A10036).

### Mice genotyping

Fetal or P7 mice were numbered and part of the toe or tail tissue was clipped for genotyping using the Mouse Direct PCR Kit (Bimake, B40013). Then, mice were genotyped by conventional PCR. For genotyping *Prmt5*^*F/F*^ mice, following oligos were used: *Prmt5-LoxP*-F, 5′-ACT GGG TTG CTC ACA ACT GC-3′ and *Prmt5-LoxP*-R, 5′-CTT CCT CTG CGT CCC ATG TTC-3′. Genotypes were determined by PCR products: 400 bp (wild-type allele) and 500 bp (flox allele). For *Emx1-Cre* mice, primer oligos were *Emx1-Cre*-F, 5′-GCC TGC ATT ACC GGT CGA TGC AAC GA-3′ and *Emx1-Cre*-R, 5′-GTG GCA GAT GGC GCG GCA ACA CCA TT-3′. The PCR products were 750 bp (Cre allele). For *Nex-Cre* mice, primer oligos were *Nex-Cre*-1, 5′-CCG CAT AAC CAG TGA AAC AG-3′ and *Nex-Cre*-2, 5′-AGA ATG TGG AGT AGG GTG AC-3′ and *Nex-Cre*-3, 5′-GAG TCC TGG CAG TCT TTT TC-3′. The PCR products included: 770 bp (Cre allele) and 525 bp (wild-type allele). For Ai9D mice, primer oligos were Ai9-WT-F, 5′-AAG GGA GCT GCA GTG GAG TA-3′, Ai14-WT-R, 5′-CCG AAA ATC TGT GGG AAG TC-3′, Ai14-Mut-F, 5′-GGC ATT AAA GCA GCG TAT CC-3′ and Ai14-Mut-R, 5′-CTG TTC CTG TAC GGC ATG G-3′. Genotypes were determined by PCR products: 297 bp (wild-type allele) and 196 bp (mutant allele). For *Trp53*^*−/−*^ mice, following oligos were used: *Trp53*-1, 5′-AGT TCT GCC ACG TGG TTG GT-3′, *Trp53*-2, 5′-GTC TCC TGG CTC AGA GGG AG-3′, and *Trp53*-3, 5′-CAG AGG CCA CTT GTG TAG CG-3′. Genotypes were determined by PCR products: 281 bp (wild-type allele) and 441 bp (mutant allele).

### Western blotting

Western blot procedures were performed as previously described [22, 23]. Briefly, mouse cortices were lysed in RIPA buffer contained SDS, boiled at 100 ℃ for 5 min, and run on 6–12% Bis–Tris gel. High-sig ECL Western Blotting Substrate (Cat# 180-5001, Tanon, China) was used for signal detection. Quantification results were obtained using Fiji/ImageJ. The primary antibodies were rabbit anti-PRMT5 (Abcam, ab109451), rabbit anti-PRMT1 (Upstate, 07-404), rabbit anti-PRMT4 (Millipore, 09-808), rabbit anti-PRMT6 (CST, 14,641), rabbit anti-PRMT7 (CST, 14762), rabbit anti-CC3 (CST, 9661), rabbit anti-γH2AX (CST, 9718), rabbit anti-RAD51 (ABclonal, A6268), rabbit anti-53BP1 (ABclonal, A5757), rabbit anti-KU70 (ABclonal, A0883), rabbit anti-ATM (ABclonal, A18257), rabbit anti-p-ATM (ABclonal, AP0008), rabbit anti-p53 (ABclonal, A3185), rabbit anti-p-p53 (ABclonal, AP0083), mouse anti-FLAG (Sigma-Aldrich, F7425), mouse anti-HA (Abcam, Ab16918), rabbit anti-H4R3me2s (ABclonal, A3159), and rabbit anti-H3R2me2s (ABclonal, A2373).

### Immunostaining

Following dissection, embryonic or postnatal brains were fixed in 4% paraformaldehyde overnight and dehydrated with 30% sucrose solution. Dehydrated samples were then embedded with O.C.T.(Tissue-Tek) and sectioned using cryostat.

For immunostaining, frozen sections are first dried and then washed 3 times with PBS buffer. Subsequently, the sections were blocked with blocking buffer containing 2% BSA and 0.2% Triton-X 100. Diluted the primary antibodies with blocking buffer and incubate the sections overnight at 4 °C. On the next day, after washing three times with PBST, the sections were incubated with fluorescence-conjugated secondary antibodies for 1.5 h. All sections were counterstained with DAPI (GENVIEW, GD3408) for 10 min. Finally, fix the coverslips to the sections with PVA.

### Nissl staining

The postnatal brains were dissected and prepared as previously described in the “Immunostaining” section. For Nissl staining, dehydrated brains were sectioned on a sliding microtome (Leica, SM2010R) at 60 μm. Sections were first washed in double-distilled water twice followed by immersion in Nissl staining solution (Beyotime, C0117) for 2 min. After staining, sections were washed with ethanol (95%) and then with ethanol (75%) 3 times.

### Open field test

Mice used for Open field test were male and were 2 months of age when the tests began. Mice of different genotypes were raised together. One hour before the start of the experiment, the mice were placed in the experimental room to acclimate to the environment. The animals were acclimatized to the testing room for 1 h before the test. Before the start of each experiment, wipe the experimental apparatus with 75% alcohol to eliminate the odor of the mice. A curtain surrounding the equipment was used to prevent interference. Then the testing mouse was placed into a 50 × 50 × 35 cm chamber and the behaviors of the mice were recorded using a camera for 15 min. The behavioral data were analyzed using an Ethovision XT 10 system (Noldus, Wageningen, The Netherlands). The chamber was cleaned between each trial.

### 5-bromo-2-deoxy-uridine (BrdU) labeling

Prepare 10 mg/mL of BrdU solution with sterile saline. Then, BrdU solution was intraperitoneally injected into pregnant female mice (50 mg/kg BrdU of the female mice body weight). 2 or 24 h later, the mice were killed, and the embryos were harvested. The brains of embryonic mice were soaked in PFA solution and fixed overnight, followed by dehydration, sectioning, and immunostaining. Antigen retrieval and HCl treatment were required for the BrdU antibody immunostaining.

### RNA-sequencing analysis

RNA was extracted from E12.5 WT and *Prmt5* cKO-Emx1 cortices using Trizol reagent (Invitrogen). RNA samples were then qualitatively tested and the mRNA library was prepared. RNA sequencing was performed by BGI company, China. Three groups of RNA samples (3 mice per genotype) were sequenced. RNA-seq reads were mapped to the mouse genome (GRCm38.p6) using HISAT2 (version 2.1.0). Differential genetic testing was performed using DESeq2 (v1.4.5) under conditions of *Q* value ≤ 0.05. GO analysis was performed using DAVID software (version 6.8) and Metascape (http://metascape.org). The raw data have been deposited in the National Center for Biotechnology Information (NCBI) with accession code PRJNA925791.

### Quantitative real-time PCR (q-PCR)

RNA was extracted from WT and *Prmt5* cKO-Emx1 embryonic cortices using TRIzol reagent (Invitrogen). The cDNA was then synthesized by reverse transcription of the RNA samples using TransScript® II One-Step gDNA Removal and cDNA Synthesis SuperMix (Transgene). Real-time PCR was performed using a Thermo Scientific PikoReal 96 Real-Time PCR System (Thermo Fisher). Cq values were obtained according the QuantStudio Design & Analysis Software (Thermo Fisher Scientific). The gene expression was normalized to the level of *Gapdh* mRNA for three biological replicates. Primer sequences used in qPCR are listed in supplemental Table 1.

### Isolation and culture of NSCs

As described in the previous study [24], the cortices of E12.5 WT and *Prmt5* cKO-Emx1 mice were isolated in Hank’s balanced salt solution (HBSS) on ice and subsequently washed twice with PBS. Then, the samples were digested with 0.05% (w/v) trypsin–EDTA (Sigma) solution. The digested samples were dissociated with a firepolished glass pipette and filtered using 70 µm cell strainers (BD Falcon, 252350). Subsequently, the isolated cells were seeded into the dishes containing the neurobasal medium with 2% B27 (Gibco, Cat No. 17504-044), 20 ng/ml basic fibroblast growth factor (bFGF) (FGF-2; PeproTech, K1606), 20 ng/ml epidermal growth factor (EGF, PeproTech, A2306), 1% antibiotics and 2 mM GlutaMAX (Gibco, 21103049). Plated cells were incubated at 37 °C with 5% CO_2_. Half of the medium was replaced every 2 days, the NSCs have digestive transfer cultured after fusion growth.

To induce DSBs using etoposide and immunostaining, NPCs cultured in vitro were plated into 24-well plates covered with clean glass slides. The next day, an appropriate amount of etoposide was added to the culture medium to a final concentration of 50 μm. After 2 h, the medium containing etoposide was replaced with fresh proliferating medium, and the cells were cultured for another 6 h. Finally, the slides were collected for immunostaining.

### DSB repair assay

We used two DNA repair detection strategies to evaluate the HR/NHEJ efficiency. For I-*Sce*I nucleases-based DR-GFP/EJ5-GFP detection system, pDRGFP (DR-GFP) (RRID: Addgene_26475) [25] or pimEJ5GFP (EJ5-GFP) (RRID: Addgene_44026) [26] plasmid and HA-I-*Sce*I plasmid were simultaneously transfected into cultured NSCs. DR-GFP contains a *SceGFP* gene and an *iGFP* gene. *SceGFP* is a modified *GFP* gene and contains an I-*Sce*I site and an in-frame termination codon. *iGFP* is a 5′ and 3′-truncated GFP gene. In the absence of I-*Sce*I nucleases, DR-GFP plasmid cannot express a functional GFP. I-*Sce*I nucleases can specifically recognized the I-*Sce*I site on DR-GFP and generate a DSB. When HR repair occurred, the DSB will be repaired from the *iGFP* gene on the same chromatid or sister chromatid, resulting in a functional *GFP* gene expression. EJ5-GFP contains a promoter that is separated from a *GFP* gene by a *puro* gene which is flanked by two I-*Sce*I sites. When I-*Sce*I nucleases exist, the puro gene will be cut off and GFP gene expressed by NHEJ repair. The proportion of GFP^+^ cells represent the efficiency of HR and NHEJ repair, respectively. PRMT5 inhibitor is added into the fresh medium 12 h after transfection. After 72 h, immunofluorescence staining was performed. The proportion of GFP^+^ cells represent the efficiency of HR and NHEJ repair, respectively.

CRISPR/Cas9-based DSB repair assay was carried out according to the previous study [27]. AAV_Efs_hSpCas9_NLS_FLAG-SV40 (RRID: addgene_97307) was used to generate the DSBs targeting the C terminus of the *Actb* coding sequence. AAV_Actb HR donor_U6_sgRNA_EF1a_GFP_polyA (RRID: addgene_97309) and AAV_Actb NHEJ donor_U6_sgRNA_EF1a_GFP_polyA (RRID: addgene_97310) were used to provide the HR donor and NHEJ donor for DSBs repair, respectively. The third generation NSCs cultured were transfected with these plasmids using Lipofectamine Stem Transfection Reagent (Invitrogen). For transfection, the ratio of Cas9 plasmid to donor plasmid is 1:1. The medium was replaced 12 h after transfection, and PRMT5 inhibitors are added into the fresh medium. After 72 h, immunofluorescence staining was performed. The percentage of mCherry^+^ cells among all the GFP^+^ cells represented the DNA repair efficiency of the cells.

### ChIP-qPCR

NSCs were seeded in multiple 10 cm dishes and PRMT5 inhibitor EPZ015666 was applied to inhibit the enzymatic activity of PRMT5. To induce DSBs, etoposide was added to the culture medium at a final concentration of 50 μm. Replace the medium containing etoposide with fresh proliferating medium after 2 h. Subsequently, NSCs were fixed with formaldehyde at a final concentration of 1%, and chromatin was prepared for ChIP-qPCR as described previously [28]. Antibodies used for ChIP assay were rabbit anti-PRMT5 (Abcam, ab109451), rabbit anti-H4R3me2s (ABclonal, A3159), rabbit anti-H3R2me2s (ABclonal, A2373), rabbit anti-H3R8me2s (ABclonal, A2374) and IgG-rabbit. Primer sequences used in the experiments are listed in Supplemental Table 2.

### Microscopy imaging and statistical analysis

Cell and tissue samples were observed and photographed with a ZEISS microscope, followed by image processing with ZEN software. For immunostained brain slice samples, the *z*-stack function was applied for laminar scanning.

For morphometric analysis, at least three pairs of animals were included for each condition in parallel experiments. To compare the differences between different layers of WT and *Prmt5* cKO cortices, we utilized DAPI staining to distinguish the subregions and layers of the brain.

For the analysis of the embryonic mouse cortices, each group of genotype samples contains at least 3 pairs of brains and is derived from two litters. For the measurement of the progenitors, the total numbers of Pax6^+^ and Tbr2^+^ cells in the VZ/SVZ were in an area of 100 μm × 200 μm (from the VZ apical surface). To compare DSBs and apoptosis, the total number of positive cells was in an area of 100 μm × 200 μm of neocortex (from the VZ apical surface).

SPSS software was used for data statistics and analysis, and Prism 8.0 (GraphPad software) was used for statistical chart drawing. As for statistics, two-tailed unpaired *t* test was used for comparison of two groups of data, and the data of three or more groups were analyzed and calculated by the one-way ANOVA method. The analyzed data was presented as the mean ± SEM. *p* < 0.05 was considered significant. Significance was marked as *p < 0.05; **p < 0.01 and ***p < 0.001.

## Results

### Deletion of *Prmt5* in dorsal telencephalic NPCs leads to microcephaly

**Fig. 1:**
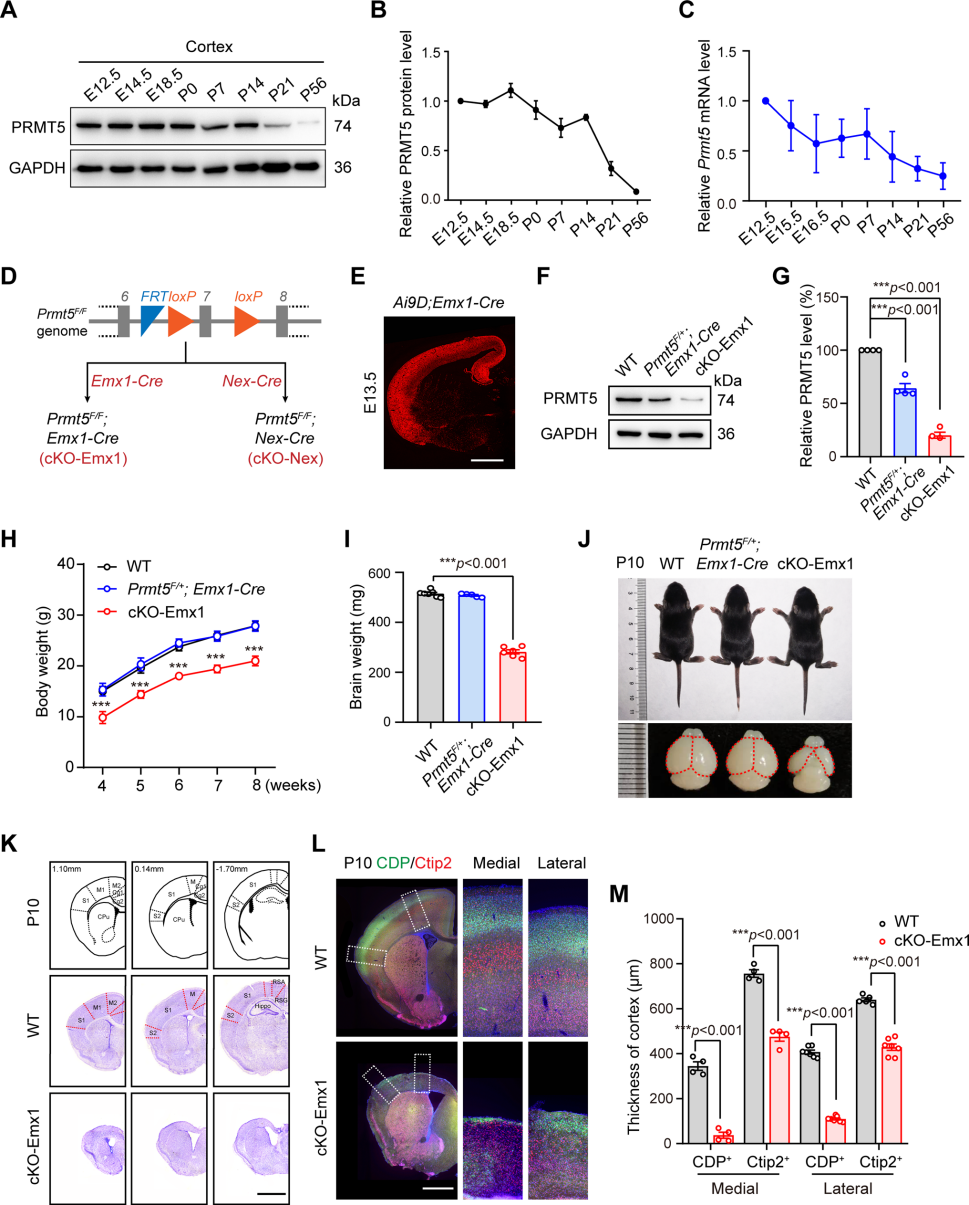
*Prmt5* deletion from NPCs leads to neuroanatomical defects in mice. **A**, **B** Western blotting and quantification analyses of PRMT5 expression in the developing cerebral cortex. Brains at different developmental stages were isolated and lysed for western blot analysis with the indicated antibodies. Expression levels were normalized to GAPDH. The expression of PRMT5 at E12.5 was set to 1. **C** Quantitative real-time PCR analyses of *Prmt5* mRNA in the developing cerebral cortex (*N* = 3, data shown as the mean ± SEM). Relative mRNA levels were normalized to *Gapdh*. The relative mRNA level of *Prmt5* at E12.5 was set to 1. **D** Schematic of *Prmt5*^*F/F*^*; Emx1-Cre* (cKO-Emx1) and *Prmt5*^*F/F*^*; Nex-Cre* (cKO-Nex) mouse generation. Exon 7 of *Prmt5* is flanked by *loxP* and will be excised by Cre recombinase. **E** Fluorescence image of E13.5 *Ai9D; Emx1-Cre* brain sections showed Emx1-driven Cre recombinase expression exclusively confined to the dorsal telencephalon. Scale bar, 500 μm. **F**, **G** Western blotting and quantification showed the expression of PRMT5 in WT, *Prmt5*^*F/*+^*; Emx1-Cre* and cKO-Emx1 cortices (P10, males; *N*  =  3 mice per genotype; data from 3 independent experiments). Expression levels were normalized to GAPDH. The ratio in WT mice was set to 100%. One-way ANOVA with Tukey’s post hoc test. **H** Body weight of WT, *Prmt5*^*F/*+^*; Emx1-Cre* and cKO-Emx1 mice at five different time points (4, 5, 6, 7 and 8 weeks after birth). One-way repeated measures ANOVA. **I** Brain weight of WT, *Prmt5*^*F/*+^*; Emx1-Cre* and cKO-Emx1 mice at P10. One-way ANOVA with Tukey’s post hoc test. **J** Representative body and brain images of WT, *Prmt5*^*F/*+^*; Emx1-Cre* and cKO-Emx1 mice at P10. **K** Nissl staining of P10 brain slices. cKO-Emx1 brains showed microcephaly, agenesis of the corpus callosum and hypoplasia of the hippocampus. Scale bar, 1 mm. **L** Representative images of CDP/Ctip2 staining of WT and cKO-Emx1 brain slices. Images on the right panel are enlarged views of the medial and lateral cortex in the dashed boxes. Scale bar, 1 mm. **M** Quantification of CDP^+^ layer and Ctip2^+^ layer thickness. One-way ANOVA with Tukey’s post hoc test. For quantification, at least 3 animals for each genotype were analyzed. Mean ± SEM; *p < 0.05; ***p < 0.001

During murine cortical neurogenesis, NPCs in the VZ maintain self-renewal through symmetrical division and form neurogenic cells through asymmetric division. The expression of PRMT5 in the cortex was maintained at relatively stable levels from E12.5 to P0, suggesting that PRMT5 may play important roles in neurogenesis (Fig. 1A–C). To address the role of PRMT5 in neurogenesis, we used *Emx1-Cre* mice to specifically knockout *Prmt5*, which is effective from E10.5 and targets neural progenitor cells (NPCs) derived from the *Emx1* lineage [29] (Fig. 1D). The expression of *Emx1*-driven Cre was exclusively confined to the dorsal telencephalon excitatory NPCs by crossing with *Ai9D* mice (Fig. 1E), which is different from *Nestin*-driven Cre that widely expressed in the entire brain [30]. The knockout efficiency was confirmed by Western blotting using E12.5 *Prmt5*^*F/F*^; *Emx1-Cre* (cKO-Emx1) cortical lysates (Fig. 1F, G). The cKO-Emx1 mice showed lighter body and brain weights (Fig. 1H–J), but survived more than one year.

We examined the brain anatomy of P10 cKO-Emx1 mice via Nissl staining. The thickness of the cKO-Emx1 cerebral cortex was reduced in graded levels along the mediolateral axis (Fig. 1K). The most severe reduction was found in the primary and secondary motor cortex (M1 and M2) and the primary somatosensory cortex (S1). In addition, ventricles were enlarged and corpus callosum and hippocampus were not observed (Fig. 1K). The cortical thickness reduction was further examined by imaging cortical lamination with layer-specific markers. The thickness of the deep layer (Ctip2^+^ cells, Tbr1^+^ cells and Foxp2^+^ cells) and superficial layer (CDP^+^ cells) were both sharply reduced (Fig. 1L, M and Fig. S1A), especially in the medial cortical area. However, these brain regions were all maintained in cKO-Nes mouse brains even though the mice displayed enlarged lateral ventricles and thinner cortex [21]. We also performed Nissl staining on adult (P56) cKO-Emx1 brain slices. The overall anatomy of P56 cKO-Emx1 brain resembled that of P10 cKO-Emx1 brain, including the complete loss of corpus callosum and hippocampal structures (Fig. S1B).

As a result of cortex malformation, adult cKO-Emx1 mice exhibited behavioral alterations with hyperactivity. The cKO-Emx1 mice traveled longer distances and displayed higher average speeds in the open field test (Fig. S1C–E).

To figure out the critical roles of PRMT5 during cortical neurogenesis, we also used *Neurod6-Cre* (*Nex-Cre*) to mediate *Prmt5* knockout in postmitotic excitatory neurons [31] (Fig. S1F–H). Results showed *Prmt5*^*F/F*^; *Nex-Cre* (cKO-Nex) mouse brains had no visible defects in brain anatomical structure or cortical cytoarchitecture (Fig. S1I–L). Altogether, PRMT5 deficiency in NPCs leads to a severe microcephaly phenotype, suggesting that PRMT5 plays a critical role in cortical development.

### PRMT5 is required for expansion of proliferative NPCs in the dorsomedial cortex

**Fig. 2:**
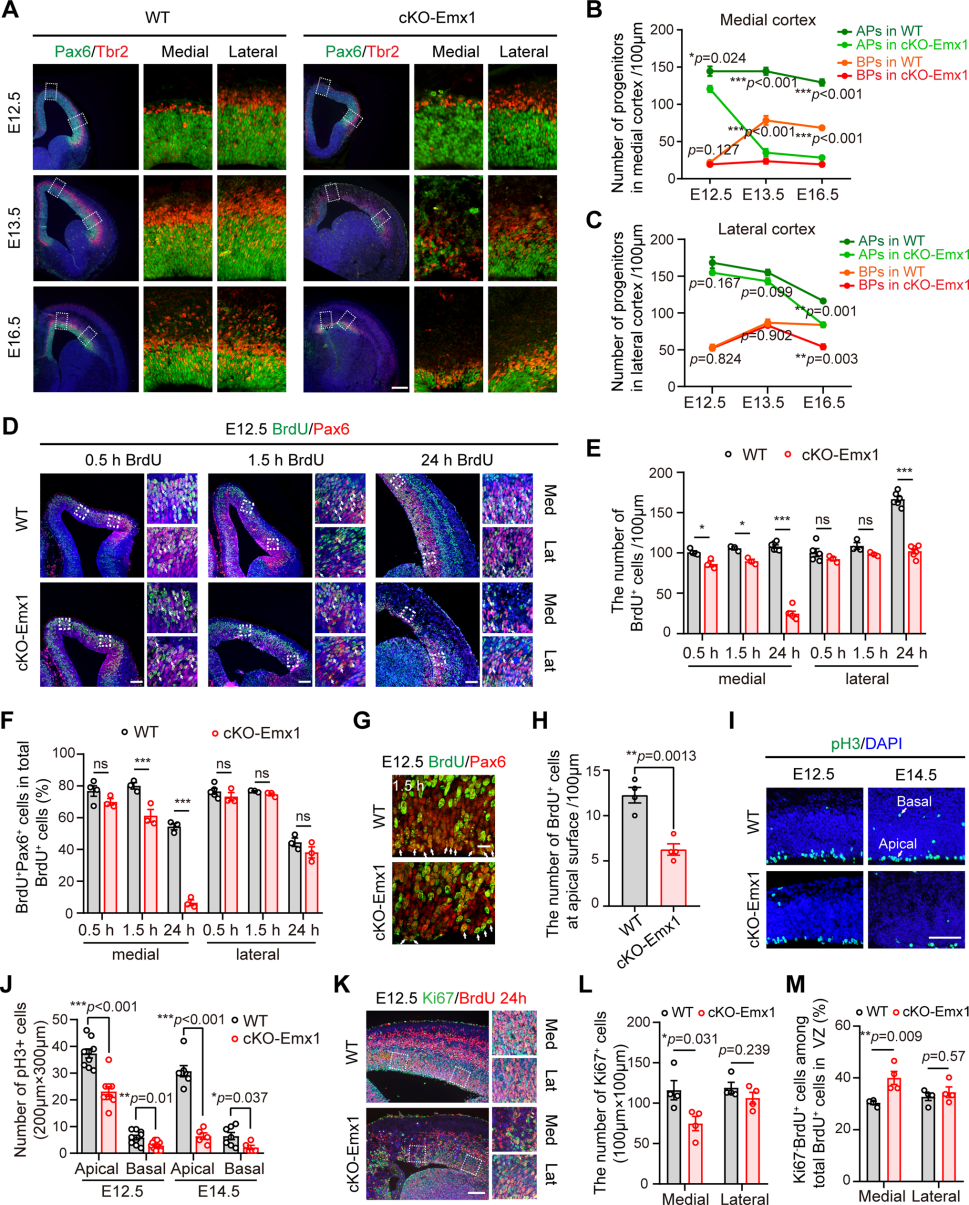
*Prmt5* deletion leads to loss of NPCs. **A** Representative image of Tbr2/Pax6 staining of WT and cKO-Emx1 brain slices. Scale bar, 200 μm. Images on the right panel are enlarged views in the dashed boxes. **B** Quantification results of Pax6^+^ APs and Tbr2^+^ BPs within a radial column of 100 μm width in the medial cortex at E12.5, E13.5, and E16.5. One-way ANOVA with Tukey’s post hoc test. **C** Quantification results of Pax6^+^ APs and Tbr2^+^ BPs within a radial column of 100 μm width in the lateral cortex at E12.5, E13.5 and E16.5. One-way ANOVA with Tukey’s post hoc test. **D** Representative image of BrdU and Pax6 staining of E12.5 WT and cKO-Emx1 brain sections after BrdU injection. E12.5 pregnant mice were administered BrdU (50 mg/kg), and the embryos were collected after 0.5 h, 1.5 h, and 24 h. Brain sections were stained with BrdU (green) and Pax6 (red) antibodies. Images on the right panel are enlarged views in the dashed boxes. Scale bar, 100 μm. **E** Quantification results of BrdU^+^ cells within a radial column of 100 μm width of the cortex. One-way ANOVA with Tukey’s post hoc test. **F** Percentage of BrdU^+^Pax6^+^ cells among total BrdU^+^ cells in WT and cKO-Emx1 cortices. One-way ANOVA with Tukey’s post hoc test. **G** Representative images of BrdU and Pax6 staining of E12.5 WT and cKO-Emx1 brain sections after BrdU injection. E12.5 pregnant mice were administered BrdU (50 mg/kg), and the embryos were collected after 1.5 h. Brain sections were stained with BrdU (green) and Pax6 (red) antibodies. Scale bar, 20 μm. **H** Quantification results of BrdU^+^ cells that reached the ventricular surface 1.5 h after BrdU injection. Two-tailed unpaired *t* test. **I** Brain sections were stained with phospho-histone H3 (pH3; green) and DAPI (blue). Representative images of pH3 staining of WT and cKO-Emx1 cortical sections are shown. Scale bar, 100 μm. **J** Quantification results of pH3^+^ cells within a 200 μm × 300 μm column at E12.5 and E14.5 in (**I**). One-way ANOVA with Tukey’s post hoc test. **K** Cell cycle exit is increased in cKO-Emx1 mice. E12.5 pregnant mice were administered BrdU (50 mg/kg) for 24 h, and the embryos were collected at E13.5. Brain sections were stained with BrdU (red) and Ki67 (green) antibodies. Scale bar, 100 μm. **L** Quantification results of Ki67^+^ cells within a column of 100 μm × 100 μm in the lateral or medial VZ of the cortex at E13.5 (*N* = 3 mice per genotype). One-way ANOVA with Tukey’s post hoc test. **M** Percentage of cell cycle exit (Ki67^−^BrdU^+^ / BrdU^+^) in the lateral or medial VZ cortex of WT and cKO-Emx1 mice (*N* = 3 mice per genotype). One-way ANOVA with Tukey’s post hoc test. For quantification, at least 3 brains for each genotype were analyzed. Mean ± SEM; *p < 0.05, **p < 0.01, ***p < 0.001

Microcephaly usually results from defects in neural progenitor pool. We, therefore, examined the apical progenitors (APs) and basal progenitors/intermediate progenitors (BPs/IPs) in the developing cortex by immunostaining Pax6 and Tbr2. A gradual decrease in APs and a rapid increase in BPs were observed in the WT brains from E12.5 to E16.5 (Fig. 2A–C). In comparison with WT mice, the Pax6^+^ cells in cKO-Emx1 mice were slightly but significantly reduced at E12.5 (Fig. 2A–C). From E13.5, we observed different phenotypes between the medial and lateral cortical areas. In the medial cortex area, Pax6^+^ cells were sharply reduced (75.8%) at E13.5, and only a minimal number of Pax6^+^ cells (21.9%) remained at E16.5 (Fig. 2A, B). The number of Pax6^+^ cells in the lateral cortex area was only moderately reduced (7.7%) at E13.5, and a substantial number of Pax6^+^ cells were still present (72.3%) at E16.5 (Fig. 2A, C). Similarly, we also observed distinctive reductions in Tbr2^+^ cells along the mediolateral axis of the cKO-Emx1 cortex. In the medial cortex, the number of Tbr2^+^ BPs more than doubled from E12.5 to E13.5 (256% increase) in WT brains, whereas Tbr2^+^ BPs slightly increased from E12.5 to E13.5 (22.4% increase) in cKO-Emx1 brain (Fig. 2A, B). In the lateral cortex, the Tbr2^+^ BPs were unchanged at E13.5 but significantly reduced at E16.5 (Fig. 2A, C). Thus, PRMT5 deficiency led to uneven AP and BP reduction along the mediolateral axis. Moreover, no detectable changes in Pax6^+^ cells and Tbr2^+^ cells were observed in cKO-Nex brains (Fig. S2A, B), which further supports the critical and specific roles of PRMT5 in NPCs, but not in differentiated neurons.

To determine whether PRMT5 deficiency impaired NPC proliferation, BrdU was administered at E12.5, and brain slices were prepared at 0.5 h, 1.5 h and 24 h post BrdU administration to trace S phase, G2/M phase and cell cycle re-entry/exit, respectively. In the medial cortex, the number of BrdU^+^ cells in the cKO-Emx1 cortex was slightly but significantly decreased at 0.5 h post BrdU administration (Fig. 2D, E). The ratio of BrdU^+^Pax6^+^ cells among total BrdU^+^ cells was similar between the WT and cKO-Emx1 cortices (Fig. 2D, F), suggesting that PRMT5-deficient NPCs had comparable DNA replication function at early S phase [32]. At 1.5 h post BrdU administration, the ratio of BrdU^+^ Pax6^+^ cells among total BrdU^+^ cells was significantly reduced in the cKO-Emx1 cortex, while 80% of BrdU^+^ cells exhibited Pax6^+^ staining in WT mice (Fig. 2D, F). At 24 h post BrdU administration, NPCs that had successfully completed a mitosis cycle entered the next cell cycle. Sharp reductions in BrdU^+^ cells and the ratio of BrdU^+^Pax6^+^ cells among total BrdU^+^ cells were present in the cKO-Emx1 cortex (Fig. 2D, F), indicating dramatic AP cell loss during this period. Only a small proportion of BrdU^+^ cells were lost during a 24-h period in the lateral cortex. No detectable changes were observed in the ratio of BrdU^+^Tbr2^+^ cells among total BrdU^+^ cells in the cKO-Emx1 cortex (Fig. S2C, D*)*. These data indicated that Pax6-positive APs were more sensitive to PRMT5 deficiency than Tbr2-positive BPs.

The amplifying apical NPCs (radial glial cells) undergo interkinetic nuclear migration in the VZ, where NPC nuclei migrate to the upper VZ for S phase and to the ventricular surface for mitosis. We found fewer BrdU^+^ cells in the cKO-Emx1 cortex reached the ventricle surface after 1.5 h of BrdU administration and reduced cellular proliferation that indicated with a mitosis marker, phospho-histone H3 (pH3) (Fig. 2G–J). Moreover, many BrdU^+^ cells displayed Ki67^−^ in the VZ of the cKO-Emx1 cortex at 24 h post BrdU administration (Fig. 2K–M), suggesting that Pax6^+^ APs exited cell cycle in PRMT5-deficient brain. These data indicated that PRMT5 deficiency impaired NPC cell proliferation and altered cell fate during embryonic neurogenesis.

Altogether, these results demonstrate that PRMT5 is essential for NPC proliferation and *Prmt5* deletion in NPCs has a major deleterious effect in the medial cortex.

### PRMT5 deficiency leads to DNA damage and apoptosis in the proliferative progenitors

**Fig. 3:**
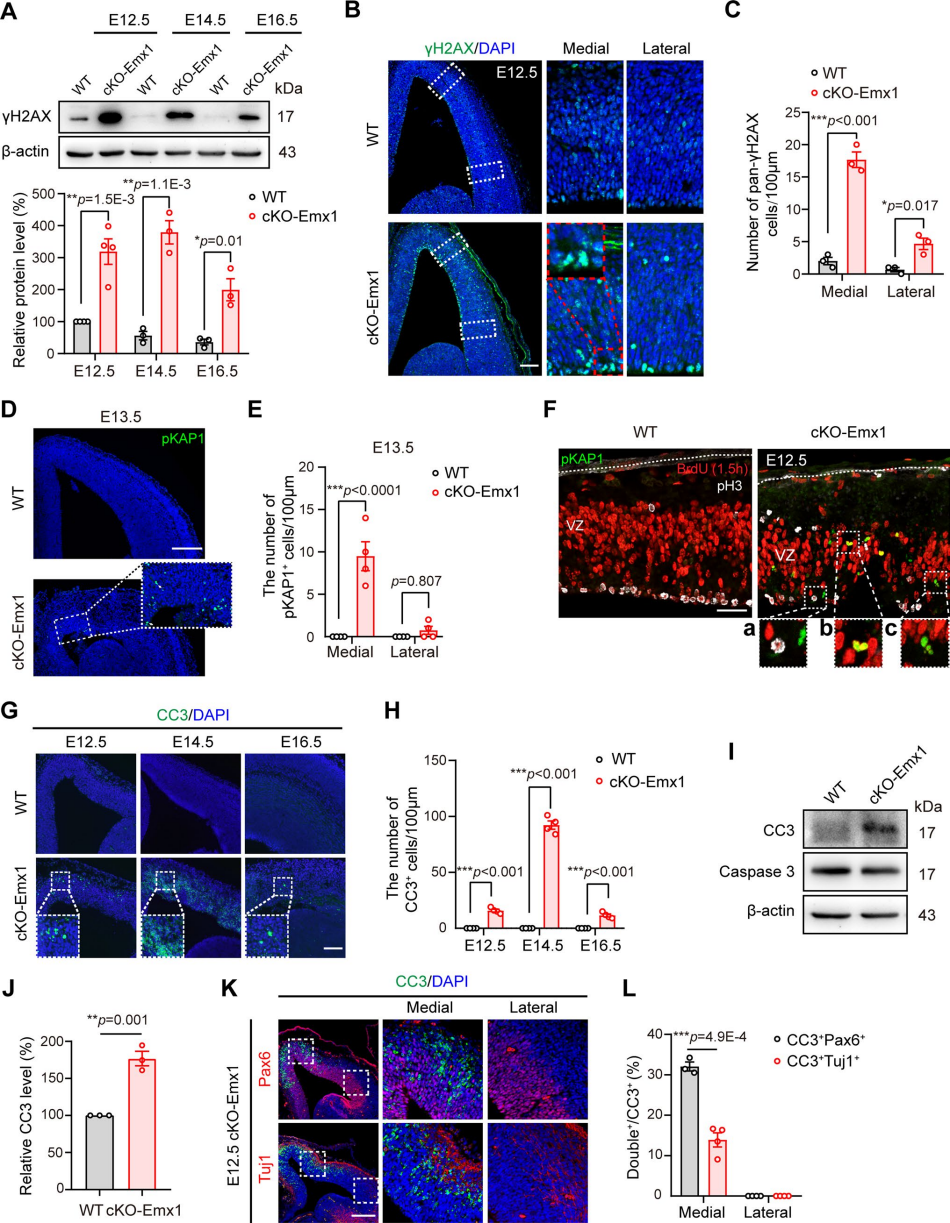
The accumulation of DSBs in NPCs ultimately leads to cell apoptosis in the cKO-Emx1 cortices. **A** Western blotting and quantification results of γH2AX levels in E12.5, E14.5, E16.5 WT and cKO-Emx1 cortices. Expression levels were normalized to β-actin. Relative protein level in E12.5 WT cortex was set to 100%. One-way ANOVA with Tukey’s post hoc test. **B** Representative images of γH2AX staining of WT and cKO-Emx1 brain slices are shown. E12.5 brain sections were immunostained with γH2AX and DAPI. Images on the right panel are enlarged views of the medial and lateral cortex in the dashed boxes. Scale bar, 100 μm. **C** Quantification of pan-γH2AX cells within a column of 100 μm width of the cortex at E12.5 from (**B**). One-way ANOVA with Tukey’s post hoc test. **D** E13.5 brain sections were immunostained with pKAP1 and DAPI. Representative images of pKAP1 staining of WT and cKO-Emx1 brains are shown. Scale bar, 200 μm. **E** Quantification results of pKAP1^+^ cells within a column of 100 μm width of the E13.5 cortex from (**D**). One-way ANOVA with Tukey’s post hoc test. **F** Analyses of S-G2 phase NPCs by 1.5 h pulsed BrdU (red), M-phase NPCs by pH3 immunostaining (white), and DSBs by pKAP1 immunostaining (green) in the E12.5 cortex. No pKAP1 staining colocalized with pH3, suggesting that NPCs with unrepaired DSBs did not progress successfully into mitosis. Scale bar, 50 μm. **A**–**C** are higher magnification views of the dashed boxes, respectively. **G** Brain sections were immunostained with the apoptosis marker cleaved caspase 3 (CC3) and DAPI. Representative images of CC3 staining of WT and cKO-Emx1 slices are shown. The insets at the bottom are higher magnification views of the dashed boxes. Scale bar, 100 μm. **H** Quantification of CC3^+^ cells within a column of 100 μm width of the cortex at E12.5, E14.5 and E16.5 from (**G**). One-way ANOVA with Tukey’s post hoc test. **I, J** Western blotting and quantification results showed that the expression levels of CC3 were upregulated in E12.5 cKO-Emx1 cortical samples. Expression levels were normalized to β-actin. The ratio of WT mice was set to 100%. Two-tailed unpaired t test. **K** Brain sections of E12.5 cKO-Emx1 were immunostained with the apoptosis markers CC3 and Pax6/Tuj1. The images on the right panel are higher magnification views of the dashed boxes. Scale bar, 100 μm. **L** Quantification results of CC3^+^Tuj1^+^ or CC3^+^Pax6^+^ cells among total CC3^+^ cells from immunostaining in (**K**). One-way ANOVA with Tukey’s post hoc test. For quantification, at least 3 brains for each genotype were analyzed. Mean ± SEM; *p < 0.05, **p < 0.01, ***p < 0.001

Proliferative progenitors have high DNA replication index particularly sensitive to replication-associated DNA damage, which include DNA double-stranded break (DSBs). Then, we explored whether PRMT5 deficiency impaired genomic integrity. Phospho-histone variant H2AX (γH2AX), which is the initial cellular responsive molecule to DSBs, was selected to indicate DSBs generation and accumulation [33, 34]. We found that γH2AX in WT brain was only evident at E12.5, which was associated with rapid NPC proliferation during early cortical development. However, γH2AX was maintained at high levels from E12.5 to E16.5 in cKO-Emx1 brains (Fig. 3A). The γH2AX-positive cells were distributed in both medial and lateral cortices. Obviously, many γH2AX-positive cells were characterized by pan-nuclear staining that mostly concentrated in the medial cortical area along the ventricular surface (Fig. 3B, C and Fig. S3A), suggesting unrepaired DNA damage [35]. Next, phospho-KAP1 (also known as TRIM28) was used to examine unrepaired heterochromatic DSBs [36]. WT mice showed no detectable phospho-KAP1 (pKAP1)^+^ cells. However, pKAP1^+^ cells in cKO-Emx1 brain were dramatically increase at the medial cortex at E13.5 (arrowheads, Fig. 3D, E and Fig. S3B), confirming unrepaired DNA damage in the cKO-Emx1 medial cortex. Remarkable, pKAP1 staining was mainly localized near the ventricular surface or in the VZ area, but pKAP1^+^ cells did not show colocalization with pH3 (Fig. 3F). These data suggest that unrepaired DSBs accumulate in PRMT5-deficient NPCs, which impair or delay the progression of the cell cycle to mitosis.

To explore the fate of these NPCs with accumulated unrepaired DSBs, we used TUNEL staining to co-label with pKAP1. We found that 100% of TUNEL^+^ cells were pKAP1-positive (data not shown), indicating that NPCs with unrepaired DSBs eventually underwent apoptosis. Furthermore, by immunostaining with the apoptosis marker cleaved caspase 3 (CC3), we found that apoptosis readily occurred in the E12.5 cKO-Emx1 cortex, and became even higher at E14.5 and still detectable at E16.5. Apoptotic cells were mainly present in the medial cortex area (Fig. 3G, H and Fig. S3C). Western blotting also verified the activation of caspase 3 in E12.5 cKO-Emx1 cortical tissue (Fig. 3I, J). In WT or *Prmt5*^*F/*+^; *Emx1-Cre* cortical slices, no DSBs accumulation and CC3^+^ cells were observed at any developmental stage (Fig. 3G, H and Fig. S3D-G).

To ascertain the cell type of apoptotic cells in the cKO-Emx1 cortex, we co-stained CC3 with Pax6 (NPCs marker) or Tuj1 (immature neurons marker). Among the total CC3^+^ cells, CC3^+^Pax6^+^ cells accounted for 32%, while CC3^+^Tuj1^+^ cells accounted for 17% (Fig. 3K, L). These results indicate that the apoptotic cells were mainly undifferentiated neural progenitor cells, and many CC3^+^ cells had already lost their cell identities. Together, these data suggest that PRMT5 deficiency leads to DSBs accumulation and subsequent cell apoptosis.

### *Prmt5* deficiency impairs the expression of genes associated with homologous recombinant repair.

**Fig. 4:**
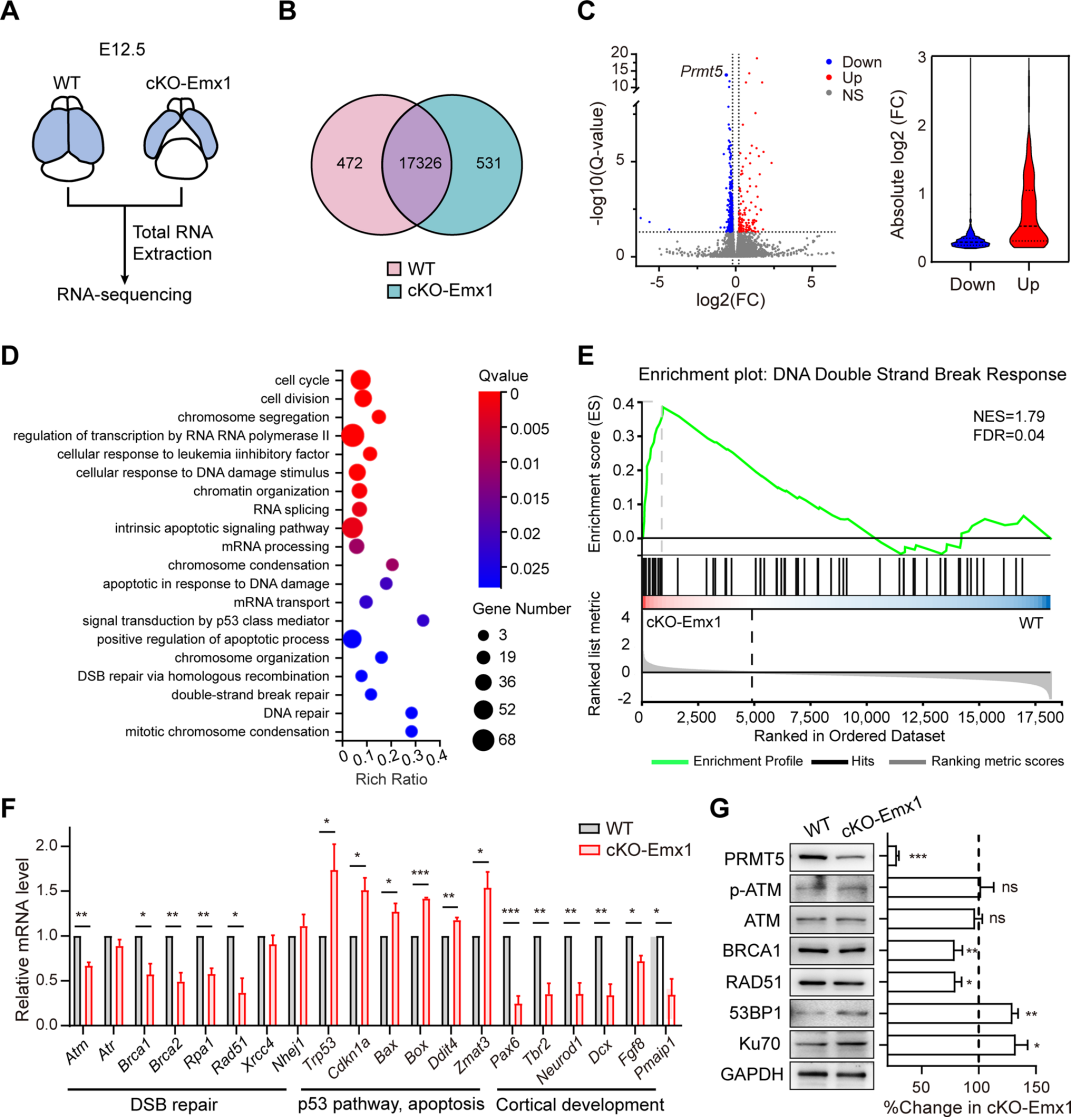
RNA-sequencing analysis shows an abnormal transcriptomic signature in the cKO-Emx1 neocortex. **A** Total RNA from E12.5 WT and cKO-Emx1 cortices were used for RNA sequencing. **B** Venn diagrams comparing genes differentially expressed in WT (pink) and cKO-Emx1 (cyan) cortices at E12.5. **C** Volcano plots illustrate differentially expressed genes between WT and cKO-Emx1 cortical samples. The number of significantly changed genes (*p*-adj < 0.05, ≥ 0.2-fold change in either direction, red dots are upregulated genes, and blue dots are downregulated genes compared to WT) is indicated. The violin plot shows the absolute change for the genes changing by  ≥ 0.2-fold (WT: *N*  =  3, cKO-Emx1: *N*  =  3). **D** GO process enrichment analysis of DEGs between WT and cKO-Emx1 samples. **E** “DNA double-strand break response” gene expression signature was tested for enrichment by GSEA in WT and cKO-Emx1 cortices. **F** Quantification of RT-qPCR results in WT and cKO-Emx1 cortices at E12.5 (WT: *N* = 3, cKO-Emx1: *N* = 3, data shown as the mean ± SEM). Genes related to “DSBs repair” and “cortical development” were downregulated, and genes related to the “p53 pathway” and “apoptosis” were upregulated in cKO-Emx1 cortices. Relative mRNA levels were normalized to *Gapdh*. The relative mRNA level in WT mice was set to 1. Two-tailed unpaired *t* test. **G** Western blotting and quantification results showed that the expression levels of HR repair-associated proteins were decreased and the expression levels of NHEJ repair-associated proteins were increased in cKO-Emx1 cortical samples (E12.5, WT: *N* = 3, cKO-Emx1: *N* = 3, data shown as the mean ± SEM). Expression levels were normalized to GAPDH. The ratio in WT mice was set to 100%. Two-tailed unpaired *t* test. For quantification, at least 3 brains for each genotype were analyzed. Mean ± SEM; *p < 0.05, **p < 0.01, ***p < 0.001

To further explore how *Prmt5* deletion results in loss of NPCs, RNA sequencing (RNA-seq) was performed to analyze the genome-wide gene expression changes in E12.5 WT and cKO-Emx1 neocortices (Fig. 4A). Approximately, 500 genes were differentially expressed when compared with control samples (Fig. 4B, C). Gene ontology analysis of biological processes (GO-P) showed that the top ranked terms included cell cycle, cell response to DNA damage, positive regulation of apoptotic process, and DSBs repair via homologous recombination (Fig. 4D). Furthermore, the upregulated genes were enriched in terms related to the apoptotic signaling pathway by p53, apoptosis response to DNA damage and microglial cell proliferation. Downregulated genes showed a significant enrichment of terms involved in the cell cycle, cell division and DNA repair and nervous system development (Fig. S4). Gene set enrichment analysis (GSEA) revealed a significant enrichment for the “DNA double-strand break response” in the cKO-Emx1 sample (Fig. 4E).

The RNA-seq results showed that genes in the “cell response to DNA damage” and “DNA repair” pathways were greatly downregulated, which is consistent with the observation of DSBs accumulation in the PRMT5-deficient mice. We tested the mRNA levels of these differentially expressed genes (DEGs) by qRT-PCR. Intriguingly, many genes that participated in HR repair processes, such as *Atm*, *Brca1*, *Brca2*, *Rpa1*, and *Rad51,* were downregulated, whereas the genes that participated in NHEJ repair, such as *Xrcc4* and *Nhej1,* were not significantly changed (Fig. 4F). Furthermore, Western blotting results also confirmed that the levels of proteins participating in HR repair were downregulated and proteins that participating in NHEJ repair were upregulated (Fig. 4G). Importantly, DEGs associated with the p53 pathway and apoptosis, such as *Trp53*, *Cdkn1a*, *Bax*, *Box*, *Ddit4*, and *Zmat3,* were upregulated, and genes related to cortical development, such as *Pax6*, *Tbr2*, *Neurod1*, *Dcx*, *Fgf8*, and *Fmaip1,* were downregulated in cKO-Emx1 cortex (Fig. 4F). Taken together, these results supported a hypothesis that the accumulation of unrepaired DSBs is due to defect of HR repair in PRMT5-deficient mice.

### p53 deletion only partially rescues the cortical defects in cKO-Emx1 mice

**Fig. 5:**
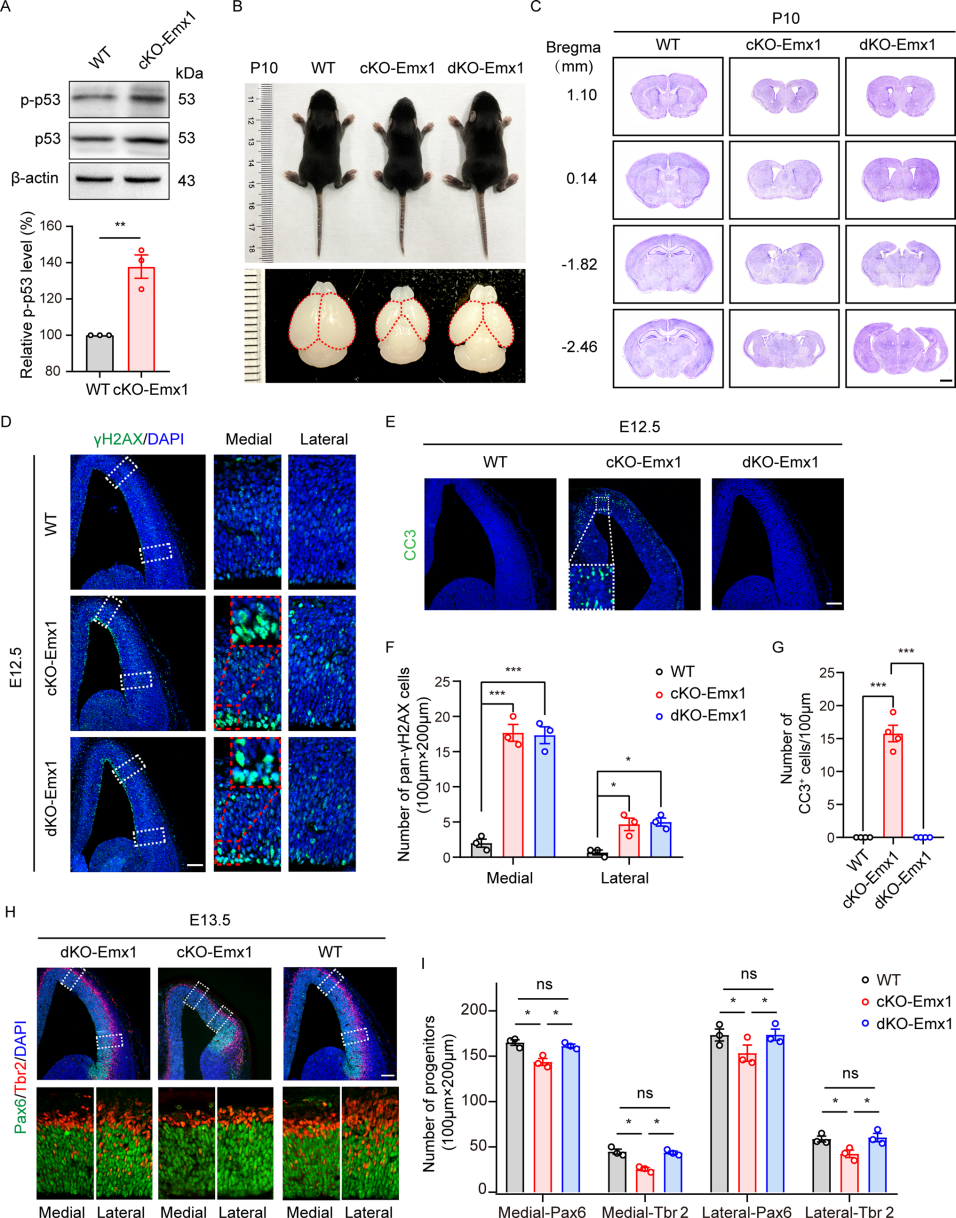
p53 deletion partially rescued the abnormal phenotypes in cKO-Emx1 brains. **A** Western blotting and quantification results showed that p53 was activated in cKO-Emx1 cortical samples (E12.5, WT: *N* = 3, cKO-Emx1: *N* = 3, data shown as the mean ± SEM). Expression levels were normalized to β-actin. The ratio in WT mice was set to 100%. Two-tailed unpaired *t* test. **B** Representative body and brain images of WT, cKO-Emx1 and dKO-Emx1 mice at P10. **C** Nissl staining of P10 WT, cKO-Emx1 and dKO-Emx1 brain slices (males; *N* = 3 mice per genotype). Scale bar, 1 mm. **D** E12.5 brain sections were immunostained with γH2AX and DAPI. Representative images of WT, cKO-Emx1 and dKO-Emx1 brain slices are shown. The right images are enlarged views of the medial and lateral cortex in the dashed boxes. Scale bar, 100 μm. **E** Brain sections were immunostained with CC3. Representative images of WT, cKO-Emx1, and dKO-Emx1 brain slices are shown. Scale bar, 100 μm. **F** Quantification results of pan-γH2AX cells within a 100 μm × 200 μm column of the cortex at E12.5 from (**D**). One-way ANOVA with Tukey’s post hoc test. **G** Quantification results of CC3^+^ cells within a column of 100 μm width of the cortex at E12.5 from (**E**). One-way ANOVA with Tukey’s post hoc test. **H** Representative images of Pax6 and Tbr2 staining of WT, cKO-Emx1 and dKO-Emx1 brain slices. Scale bar, 100 μm. Images on the right panel are enlarged views in the dashed boxes. **I** Quantification results of Pax6^+^ APs and Tbr2^+^ BPs within a radial column of 100 μm width in the medial or lateral cortex from (**H**). One-way ANOVA with Tukey’s post hoc test. For quantification, at least 3 brains for each genotype were analyzed. Mean ± SEM; *p < 0.05, **p < 0.01, ***p < 0.001

Normally, p53 is activated in response to DNA damage and drives the expression of downstream targets that mediate cell cycle arrest, DNA repair, or apoptosis [37, 38]. It has been known that PRMT5 is a co-factor of p53 that responds to DNA damage [39]. Our RNA-sequencing results showed that apoptotic signaling mediated by the p53 pathway was activated. Indeed, apoptotic signaling mediated by p53 was activated in the cKO-Emx1 cortex, which was confirmed by western blotting (Fig. 5A) and is consistent with our observation of cell apoptosis in the cKO-Emx1 cortex. We tried to explore whether suppression of p53 could rescue the microcephaly phenotype in cKO-Emx1 mice. Thus, we generated *Prmt5*^*F/F*^; *Emx1-Cre*; *Trp53*^−/−^ (dKO-Emx1) mice. The dKO-Emx1 mice were viable and had a regular body size. However, the brains size and anatomy were still significantly different from those of WT mice (Fig. 5B, C). The cortical thickness of dKO-Emx1 mice was thinner than that of WT mice, and the corpus callosum and hippocampus severely malformed (Fig. 5C). Moreover, part of the cortex along the mediolateral axis was still missing, which resembled the defect of the cKO-Emx1 brain.

We further investigated the phenotypes of DSBs accumulation and cell apoptosis in the dKO-Emx1 cortex. There was still substantial pan-γH2AX nuclear staining in the medial cortices (Fig. 5D, F) but no detectable apoptosis (indicated by CC3) (Fig. 5E, G). Distinctly, deletion of p53 recover both apical and basal progenitors in the dKO-Emx1 cortex at E12.5 (Fig. 5H, I). Thus, these data suggest that p53-dependent apoptosis was a consequence of DSBs accumulation in cKO-Emx1.

### PRMT5 deficiency disrupted homologous recombination DNA repair in NPCs

**Fig. 6:**
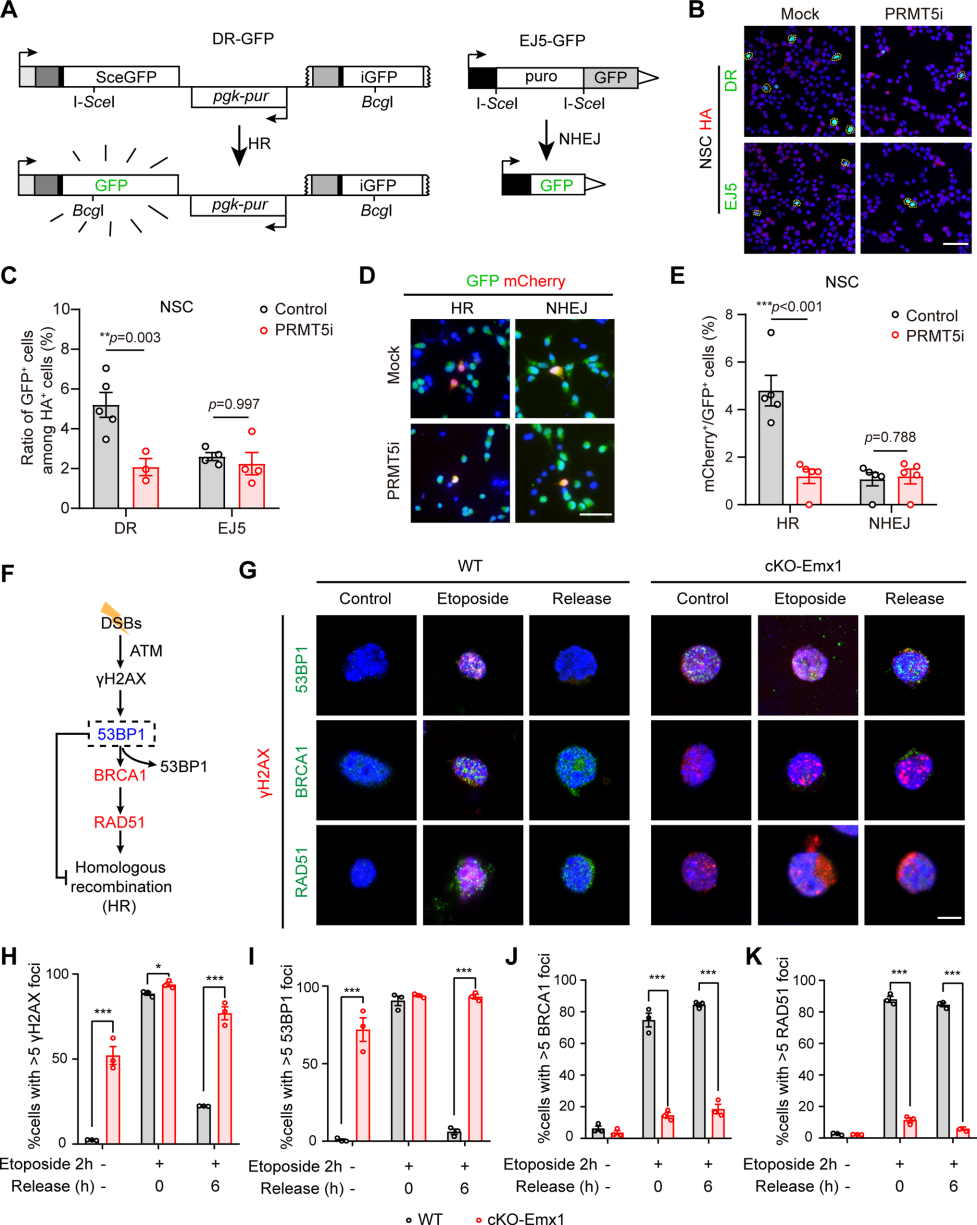
*Prmt5* deletion from NSCs disrupts homologous recombination DNA repair. **A** Schematic illustration of in vitro DSBs repair assay using DR-GFP/EJ5-GFP detection system. DR-GFP contains a *SceGFP* gene and an *iGFP* gene. *SceGFP* is a modified *GFP* gene and contains an I-*Sce*I site and an in-frame termination codon. *iGFP* is a 5′ and 3′-truncated GFP gene. I-*Sce*I nucleases specifically recognized the I-*Sce*I site on DR-GFP and generate a DSB. When HR repair occurred, the DSB will be repaired from the *iGFP* gene on the same chromatid or sister chromatid, resulting in a functional *GFP* gene expression. EJ5-GFP contains a promoter that is separated from a *GFP* gene by a *puro* gene which is flanked by two I-*Sce*I sites. When I-*Sce*I nucleases exist, the puro gene will be cut off and GFP gene expressed by NHEJ repair. **B** Representative fluorescence staining images of DMSO (Mock) or PRMT5 inhibitor (PRM5i) treated NSCs after transfection for 72 h. Scale bar, 40 μm. **C** Quantification of the percentage of GFP^+^ cells among total transfected HA^+^ cells from (**B**). Two-tailed unpaired *t* test. **D** Representative fluorescence staining images of DMSO (Mock) or PRMT5 inhibitor (PRM5i) treated NSCs after transfection for 72 h. Scale bar, 20 μm. **E** Quantification of the percentage of mCherry^+^GFP^+^ cells among all the GFP^+^ cells from (**D**). **F** Schematic of DNA double-strand break repair. **G** Representative images of γH2AX (red), 53BP1 (green, upper), BRCA1 (green, medial) and RAD51 (green, lower) immunostaining results during the DNA repair process. WT and cKO-Emx1-derived NSCs were treated with 50 μm etoposide for 2 h and then incubated with fresh medium for the times indicated. Representative images are shown. Scale bar, 5 μm. **H–K** Quantification of the percentage of cells with > 5 γH2AX foci (**H**), cells with > 5 53BP1 foci (**I**), cells with > 5 BRCA1 foci (**J**), cells with > 5 RAD51 (**K**) foci from (**G**). One-way ANOVA with Tukey’s post hoc test. Mean ± SEM; *p < 0.05, **p < 0.01, ***p < 0.001

To figure out the necessity of PRMT5 in DNA repair, we used a canonical DNA repair detection system, DR-GFP and EJ5-GFP [25, 26], to examine the effect of *Prmt5* deletion on DSBs repair pathway in NSCs. In this strategy, the mutated GFP would resume expression after HR or NHEJ repair. Therefore, the proportion of GFP^+^ cells among total transfected cells represent the efficiency of HR and NHEJ repair (Fig. 6A). Results showed the ratio of HR repair was 5% in control NSCs, which was comparable to the HR repair efficiency reported in a previous literature [27], but reduced to only 2% in NSCs treated with a potent and selective PRMT5 inhibitor, EPZ015666 [40]. In contrast, PRMT5 inhibition had no significant effect on NHEJ repair (Fig. 6B, C). We also applied this strategy to examine the DSBs repair efficiency in HEK293T cells. Again, PRMT5 inhibition only impaired HR repair efficiency but not NHEJ repair efficiency in HEK293T cells. (Fig. S5A, B).

To further confirm the specific effect of PRMT5 inhibition on HR, we employed a CRISPR-Cas9 based strategy to discriminate HR or NHEJ repair in in vitro*-*cultured PRMT5-deficient NSCs [27, 41]. As shown in Fig. S5C, Cas9 protein specifically cleaved the reading frame of the *Actb* gene via a sgRNA guidance manner. The subsequent repair of DSBs through either HR or NHEJ would introduce a mCherry reading frame into the genome (Fig. S5C). The HR or NHEJ repair efficiency was calculated by examining the mCherry^+^GFP^+^ NSCs among total GFP^+^ NSCs. The results showed that inhibition of PRMT5 only affected the HR repair efficiency in NSCs (Fig. 6D, E). The similar results were also obtained from HEK293T cells that treated with PRMT5 inhibitor (Fig. S5D, E). Altogether, these results supported a selective impairment of HR in PRMT5-deficient NSCs.

To further delineate the role of PRMT5 in DNA repair, we induced DSBs using etoposide, a topoisomerase II inhibitor (a DNA damage agent), in cultured primary neural stem cells (NSCs) isolated from E12.5 WT cortex. Results showed that etoposide induced DSBs generation in a dose-dependent manner indicated by γH2AX expression (Fig. S5F, G). A concentration of 50 μm etoposide induced a moderate level of DSBs. As expected, the expression level of PRMT5 was elevated significantly in response to DSBs (Fig. S5F, G). After 6 h of etoposide removal, most DSBs were repaired, and PRMT5 still remained at a high level (Fig. S5H–K). These results supported that PRMT5 participate in DNA repair in response to DSBs accumulation.

In the initial phase of HR repair, 53BP1 and BRAC1 play pivotal antagonistic roles, that is BRCA1 phosphorylated by ATM to form a DNA repair complex which competes with 53BP1 (Fig. 6F) [42]. Using localization of BRCA1 and RAD51 as an indicator of HR activity, we found that the ratios of cells with > 5 positive foci of γH2AX, 53BP1, BRCA1 and RAD51 were dramatically elevated after etoposide incubation in WT NSCs. After removal of etoposide, the numbers of cells with γH2AX and 53BP1 were decrease (Fig. 6G–). And the cells with BRCA1 and RAD51 foci were still maintained at a high level, indicating an effective HR repair in the NSCs (Fig. 6G, J, K). In contrast, the PRMT5-deficient NSCs showed a constitutively high ratio of γH2AX- and 53BP1-positive cells even with no etoposide incubation, which was consistent with the high γH2AX and pKAP1 staining in vivo. The ratios of cells with BRCA1 and RAD51 foci were only 5–10% in *Prmt5*-deficient cells upon etoposide induction, indicating that the HR repair pathway failed to respond to DNA damage in these NSCs (Fig. 6G–K). Thus, the DSBs were not repaired neither in PRMT5-deficient condition nor in etoposide treatment condition.

Altogether, these data suggested that the accumulation of DSBs in cKO-Emx1 NSCs result from impaired HR repair.

### PRMT5 specifically catalyzed H3R2me2s to regulate HR during G2/M phase in NPCs

**Fig. 7:**
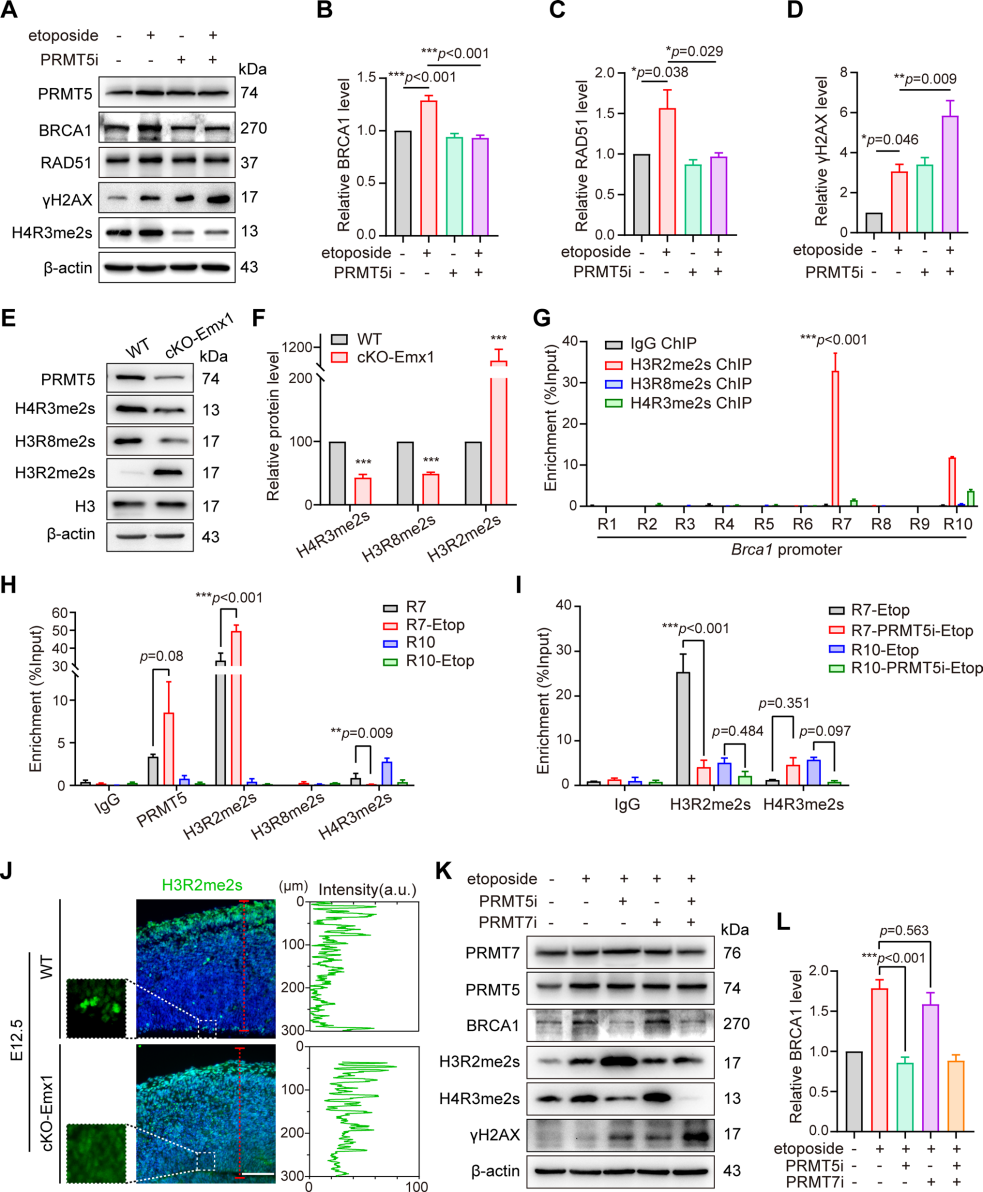
PRMT5 regulated expression of HR-related genes via histone methylation during DNA repair process. **A** Western blotting showed that PRMT5 inhibition abolished BRCA1 and RAD51 upregulation in response to etoposide treatment. **B–D** Quantification of BRCA1 (**B**), RAD51 (**C**) and γH2AX (**D**) levels via Western blotting from (**A**), respectively. Expression levels were normalized to β-actin. One-way ANOVA with Tukey's post hoc test. **E** Western blotting showed that the levels of H4R3me2s and H3R8me2s were decreased and that the levels of H3R2me2s were increased in cKO-Emx1 cortical samples. **F** Quantification results of Western blotting from (**E**) (E12.5, WT: *N* = 3, cKO-Emx1: *N* = 3). Expression levels were normalized to β-actin. The ratio in WT mice was set to 100%. Two-tailed unpaired *t* test. **G** Quantification of ChIP-qPCR results between each *Brca1* promoter region with H3R2me2s, H3R8me2s and H4R3me2s in NSCs. For each biological replicate, the value for IP was normalized to the value for IgG to calculate the fold change. Two-tailed unpaired *t* test. **H** Quantification of ChIP-qPCR results between the R7 and R10 Brca1 promoter regions with the indicated proteins (PRMT5, H3R2me2s, H3R8me2s and H4R3me2s) in NSCs with or without etoposide treatment. For each biological replicate, the value for IP was normalized to the value for IgG to calculate the fold change. One-way ANOVA with Tukey's post hoc test. **I** Quantification of ChIP-qPCR results between the R7 and R10 *Brca1* promoter regions with the indicated proteins (H3R2me2s, and H4R3me2s) in NSCs with or without PRMT5 inhibitor treatment. For each biological replicate, the value for IP was normalized to the value for IgG to calculate the fold change. One-way ANOVA with Tukey's post hoc test. **J** E13.5 brain sections were immunostained with H3R2me2s antibody and DAPI. Representative images of WT and cKO-Emx1 brain slices are shown. The left images are enlarged views in the dashed boxes. Scale bar, 100 μm. The right images are the quantification of the fluorescence intensity of H3R2me2s staining on the red dashed line in the cortex. **K, L** Western blotting and quantification analyses of BRCA1 levels under different treatment conditions in cultured NSCs. Expression levels were normalized to β-actin. One-way ANOVA with Tukey’s post hoc test. For quantification, data were from at least 3 independent experiments. Mean ± SEM; *p < 0.05, **p < 0.01, ***p < 0.001

The expression levels of HR-related genes strictly related to HR repair processes [7]. In our RNA-seq results, many genes that involved in HR were downregulated in the developing cKO-Emx1 cortex. Thus, we speculated that the insufficient expression of HR-related genes failed to respond to DNA damages in PRMT5-deficient cortex. As expected, PRMT5 inhibition or specific deletion both abolished BRCA1 and RAD51 upregulation in response to etoposide treatment, and both resulted in high level of γH2AX (Figs. 7A–D and S6A–C). Thus, these data suggested that the transcription regulation of HR-related genes during DNA damage relies on PRMT5 function.

As a protein arginine methyltransferase, PRMT5 has been shown to regulate gene transcription by regulating histone methylation [14]. Indeed, the methylation levels of H3R2me2s, H3R8me2s and H4R3me2s, which are specific modification sites of PRMT5, were all significantly altered in cKO-Emx1 cortical samples (Fig. 7E, F). It is still unclear how exactly PRMT5 regulates the expressions of HR-related genes. Consistent with previous report [16], PRMT5 was reported to bind to the promoter region of *Brca1*, *Brca2*, *Rad51*, *Rad51ap1* and *Rad51d* (Fig. S6D), indicating a potential transcription regulation network.

We then chose BRCA1 as a target to further delineate the molecular mechanism of PRMT5 in regulating HR-related gene expression. We designed ten pairs of primers to cover the *Brca1* upstream region from the transcription start site (TSS) to the −2000 bp site (Fig. S6E). We found that Region 7 (R7) of *Brca1* promoter was most significantly enriched with PRMT5 (Fig. S6F). The R7 region of *Brca1* promoter only bound with H3R2me2s, but not H3R8me2s or H4R3me2s (Fig. 7G). More importantly, upon etoposide treatment, the binding between PRMT5 and R7 of the *Brca1* promoter was significantly elevated (Fig. 7H). The binding affinity between R7 region of *Brca1* and H3R2me2s was also significantly elevated in response to DSBs (Fig. 7H). These data suggested a mechanism that PRMT5-catalyzed H3R2me2s promotes BRCA1 expression in response to DNA damage. Intriguingly, the overall expression level of H3R2me2s was elevated after PRMT5 inhibition, but the enrichment of H3R2me2s at *Brca1* promoter R7 region was significantly reduced (Fig. 7I). Surprisingly, we found PRMT5 deletion specifically abolished H3R2me2s at the ventricle surface of VZ, where NPCs underwent G2/M phase (Fig. 7J). To answer why PRMT5 had a specific effect on H3R2me2s at the ventricle surface of VZ, we then examined the distribution of H3R2me2s, PRMT5 and PRMT7, which is another methyltransferase that acts on H3R2me2s, in the developing mouse brain. Though PRMT5 and PRMT7 both showed ubiquitous distribution in the developing mouse brain, only PRMT5 displayed a cytoplasmic-to-nuclear translocation in pH3^+^ cells, which were also the H3R2me2s-positive cells (Fig. S6H). Such cytoplasmic-to-nuclear translocation of PRMT5 also happened in cultured HEK293T cells, while PRMT7 did not show enhanced distribution in the nucleus during G2/M phase (Fig. S6G). These data suggested that only PRMT5 catalyzed H3R2me2s during G2/M phase in NPCs, and further proved why the function of PRMT5 is only critical for proliferating NPCs. In support of this hypothesis, selective inhibition of PRMT7 significantly reduced the total level of H3R2me2s, but did not impair *Brca1* upregulation during DNA damage (Fig. 7K, L).

Considering the critical role of BRCA1 in initiating HR repair, we tried to rescue DSBs accumulation in PRMT5-deficient NSCs by overexpressing BRCA1. When PRMT5 was inhibited, most NPCs could not undergo DNA repair after etoposide treatment, which was manifested by enhanced 53BP1 foci, decreased RAD51 expression, and activation of apoptosis. Overexpressing BRCA1 in NSCs not only reduced the accumulation of DSBs but also significantly restored RAD51 foci and expression level in the nucleus. As expected, overexpressing BRCA1 remarkably attenuated the apoptosis of NSCs (Fig. S7A–H).

**Fig. 8:**
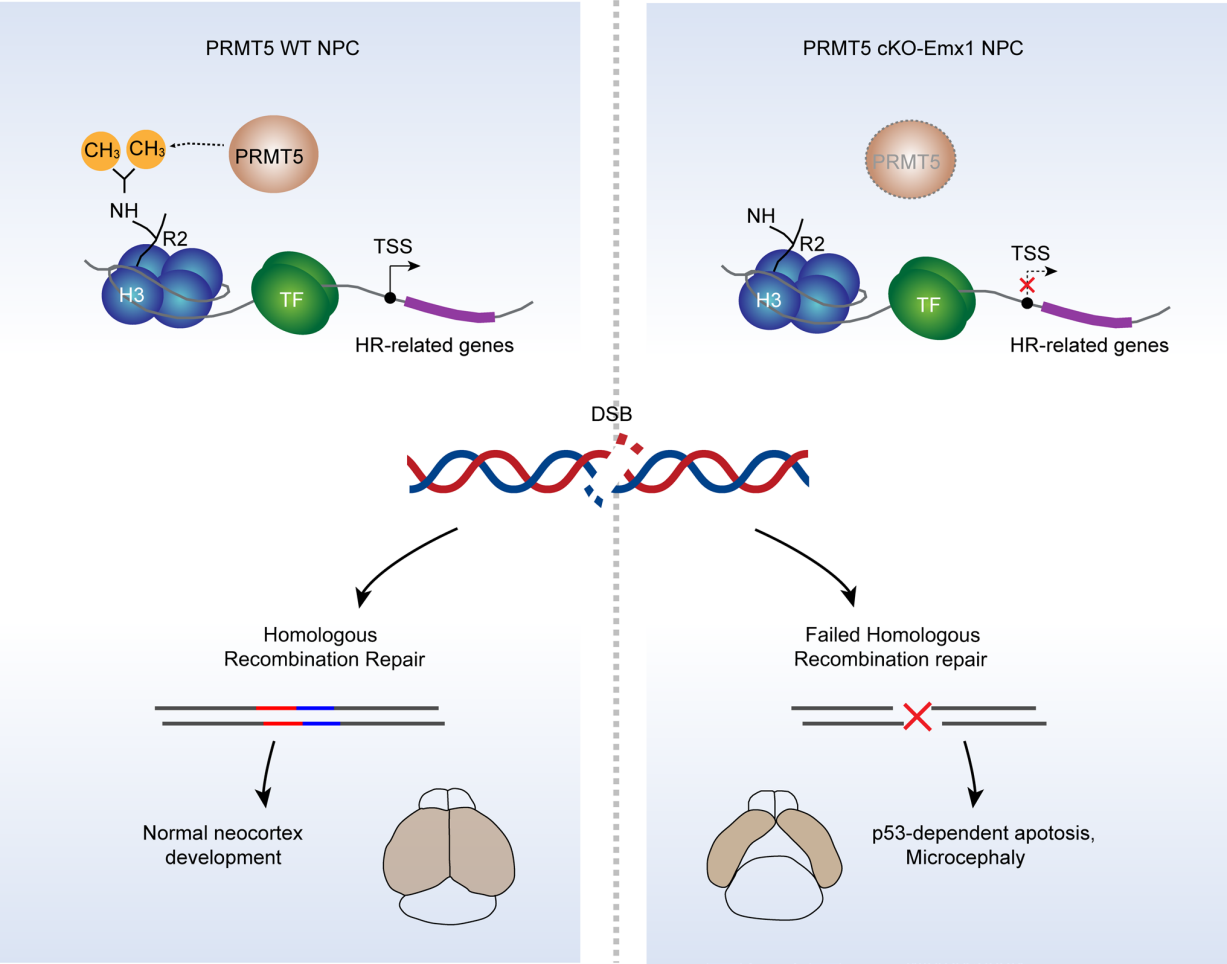
Graphical abstract showing PRMT5 is essential to maintain the genomic integrity of NPCs during neocortex development. PRMT5 regulates the transcription of HR-related genes by catalyzing the dimethylation modification of H3R2. In PRMT5-deficient NPCs, homologous recombination repair fails to respond to DSBs and resulted DSBs accumulation. NPCs with unrepaired DSBs will undergo p53-dependent apoptosis, which ultimately leads to microcephaly in mice

Collectively, our data suggest that PRMT5-mediated H3R2me2s is critical to promote HR-related gene expression in proliferating NPCs. PRMT5 deletion impairs HR repair in response to DNA damage. Such defects eventually lead to the accumulation of DSBs and p53-dependent cell death (Fig. 8).

## Discussion

The dorsal telencephalon is developed from progenitors that are derived from the VZ and SVZ. VZ progenitors undergo proliferation, differentiation, and migration to reach the cortical plate. In this study, specific knockout of PRMT5 from the beginning of neurogenesis resulted in reduced cortical thickness. These results demonstrate the necessity of PRMT5 in the proliferation of NPCs during cortical development, which is consistent with the effect of PRMT5 on cell proliferation in embryonic pluripotent cells [11], NSCs [12], oligodendrocytes [43] and immune cells [44, 45]. PRMT5 conditional knockout mice also exhibited defects in the formation of the corpus callosum and hippocampus. These phenotypes can result from the loss of cortical neurons, which was due to PRMT5 deficiency in NPCs. Together, these results identify PRMT5 as a critical regulator that preferentially preserves the proliferation capacity of NPCs.

DNA double-strand breaks (DSBs) occur during NPC proliferation, which is one of the biggest challenges during embryonic brain development. The choice of DSBs repair strategy and the fidelity of DNA repair seriously affect the survival, proliferation, and differentiation of NPCs, which in turn affects the development of the cortex. In this study, we show that PRMT5 deletion in NPCs led to accumulation of unrepaired DSBs, which triggered p53-dependent cell apoptosis and massive loss of NPCs during neocortical development. We also observed a role of PRMT5 in maintaining NPC genomic integrity by regulating the expression of HR-related genes. Although some studies have shown the importance of PRMT5 for maintaining genomic stability and DNA repair in in vitro-cultured cells [16–19, 46], it is still unknown whether PRMT5 is involved in the DNA repair process in NPCs during cortical development. Our data demonstrated that PRMT5 and the symmetric H3R2 dimethylation catalyzed by PRMT5 have unique functions in proliferating NPCs. Deficiency of PRMT5 specifically abolished H3R2me2s at the ventricle surface of VZ, which resulted DSBs accumulation and high KAP1 phosphorylation at this region. Eventually, such NPCs cannot go through the cell cycle and lead to cell apoptosis. To our surprise, only PRMT5-catalyzed H3R2me2s is responsible for *Brca1* upregulation during DNA damage response. PRMT7, which can also catalyze H3R2me2s, has no role in regulating *Brca1* expression. So far, the function of PRMT7 in DNA damage response is still obscure and PRMT7-deficient mice did not show microcephaly [47, 48]. Thus, H3R2 dimethylation that catalyzed by different enzymes exert distinctive functions in the genome. Such phenomenon has been reported for H4R3me2s [49, 50]. The function of PRMT7-catalyzed H3R2me2s in the developing brain needs to be further explored. We also noticed that there was an increase of H3R2me2s in the CP of PRMT5-deficient brains. As we showed both PRMT5 and PRMT7 expressed in this region, we speculate that there is a functional balance between PRMT5 and PRMT7. The deletion of PRMT5 may break the balance and enhance the function of PRMT7. In addition, other studies have suggested that PRMT5 regulates HR repair by indirectly regulating the level of acetylation of histone H4, such as the variable splicing of acetyltransferase TIP60 [17], or by regulating methylation of the TIP60 co-factor RUVBL1 in mouse fetal liver hematopoietic cells [18]. However, in our results, we did not observe any changes in TIP60 expression or splicing in cortical samples of cKO-Emx1 mice, nor did we find an interaction between PRMT5 and TIP60 (data not shown), presumably due to different tissue types and developmental stages. Nevertheless, our data clearly suggested that PRMT5 regulates HR-related gene expression via modulating histone arginine methylation.

PRMT5 depletion has been reported to trigger aberrant splicing of *Mdm4*, a key p53 activator in NSCs/NPCs [21]. In our results, we also found that p53 was significantly activated after PRMT5 deletion in NPCs. However, codeletion of *Trp53* with *Prmt5* did not completely rescue the phenotype that appeared in cKO-Emx1 mice. There were still a large number of DSBs accumulated in dKO-Emx1 cortices. In contrast, the accumulation of DNA damage was significantly recovered after overexpression of BRCA1, an important molecule in HR repair. Taken together, these results suggest that the activation of p53 signaling in PRMT5-deficient NPCs is a downstream result in response to unrepaired DNA damage.

Surprisingly, the effects of PRMT5 deficiency are heterogeneous in the developing brain. Our analysis of PRMT5 cKO-Emx1 mice revealed that unrepaired DSBs and apoptosis focused in medial cortical but not in lateral cortical NPCs. However, our staining results showed the distribution of PRMT5 was quite uniform in the VZ and SVZ of the medial and lateral cortex, which was similar to a previous report using a different PRMT5 antibody [12]. During cortical development, NPCs would firstly undergo symmetrical division and then shift to asymmetrical division. The symmetrical divided NPCs prefer HR repair because they have longer S phase that ensure the success of completion of HR repair. In contrast, the asymmetrical divided NPCs normally apply NHEJ repair [51]. Studies have shown that NPCs in the lateral cortex transform to asymmetrical division earlier [52, 53]. In E12.5 mouse cortex, the majority of NPCs in lateral cortex already expressed NEUROG2, which is the marker of asymmetrical division [41]. Thus, it explained why PRMT5 deficiency had a stronger deleterious effect at the medial cortex region, because much more NPCs in this region required HR to repair the DNA damage during proliferation. In contrast, NPCs in the lateral cortex could use NHEJ for DNA repair, which didn’t significantly affect by PRMT5 deletion. Similar phenotype had been reported in mouse brains that lack BRCA1 [54], BRCA2 [41], TOPBP1 [55] and INO80 [41]. All these proteins are key regulators of HR in proliferating NPCs. Taking INO80 as an example, as a key regulator of HR repair, *Ino80* deletion leads to unrepaired DNA breaks and apoptosis of symmetrical divided NPCs, but not in asymmetrical divided NPCs. As a result, the medial corticogenesis was disrupted, while the lateral corticogenesis maintained. Overall, our results support that the DSBs repair strategy of an NPC correlated with phenotypic severity in PRMT5 deficiency condition.

In conclusion, our findings demonstrate that PRMT5-catalyzed H3R2me2s plays unique roles in regulating HR-related gene expression in proliferating NPCs. Thus, PRMT5 is essential to maintain the genomic integrity of NPCs during neocortex development.

### Supplementary Information

Below is the link to the electronic supplementary material.

Supplementary file1 (DOCX 26081 KB)

## Data Availability

The raw data for RNA sequencing can be assessed at NCBI with accession number PRJNA925791. All the other data supporting the findings of this study are available from the corresponding authors upon request.
