# Supplementary material for: PRMT5-mediated homologous recombination repair is essential to maintain genomic integrity of neural progenitor cells
Source: Cell Mol Life Sci. 2024 Mar 8;81(1):123. doi: 10.1007/s00018-024-05154-x (PMC10923982; doi:10.1007/s00018-024-05154-x)
Supplement: Supplementary file 1 — Supplementary file1 (DOCX 26081 KB) [file 18_2024_5154_MOESM1_ESM.docx]

**Supplementary Material**

**PRMT5-mediated homologous recombination repair is essential to maintain genomic integrity of neural progenitor cells**

Ya-Jun Wang^1^, Jian-Bo Cao^1^, Jing Yang^1^, Tong Liu^1^, Hua-Li Yu^1^, Zi-Xuan He^1^, Shi-Lai Bao^2^, Xiao-Xiao He^1*^, Xiao-Juan Zhu^1^^*^

^1^Key Laboratory of Molecular Epigenetics, Ministry of Education, Institute of Genetics and Cytology, Northeast Normal University, Changchun 130024, China.

^2^State Key Laboratory of Molecular and Developmental Biology, Institute of Genetics and Developmental Biology, Chinese Academy of Sciences, Beijing 100101, China.

**^*^Correspondence author**

Email: [zhuxj720@nenu.edu.cn](mailto:zhuxj720@nenu.edu.cn); [hexx100@nenu.edu.cn](mailto:hexx100@nenu.edu.cn).

This file contains the following:

Supplemental figure S1-S7 and their legends

Supplemental table S1, S2 and their legends

**
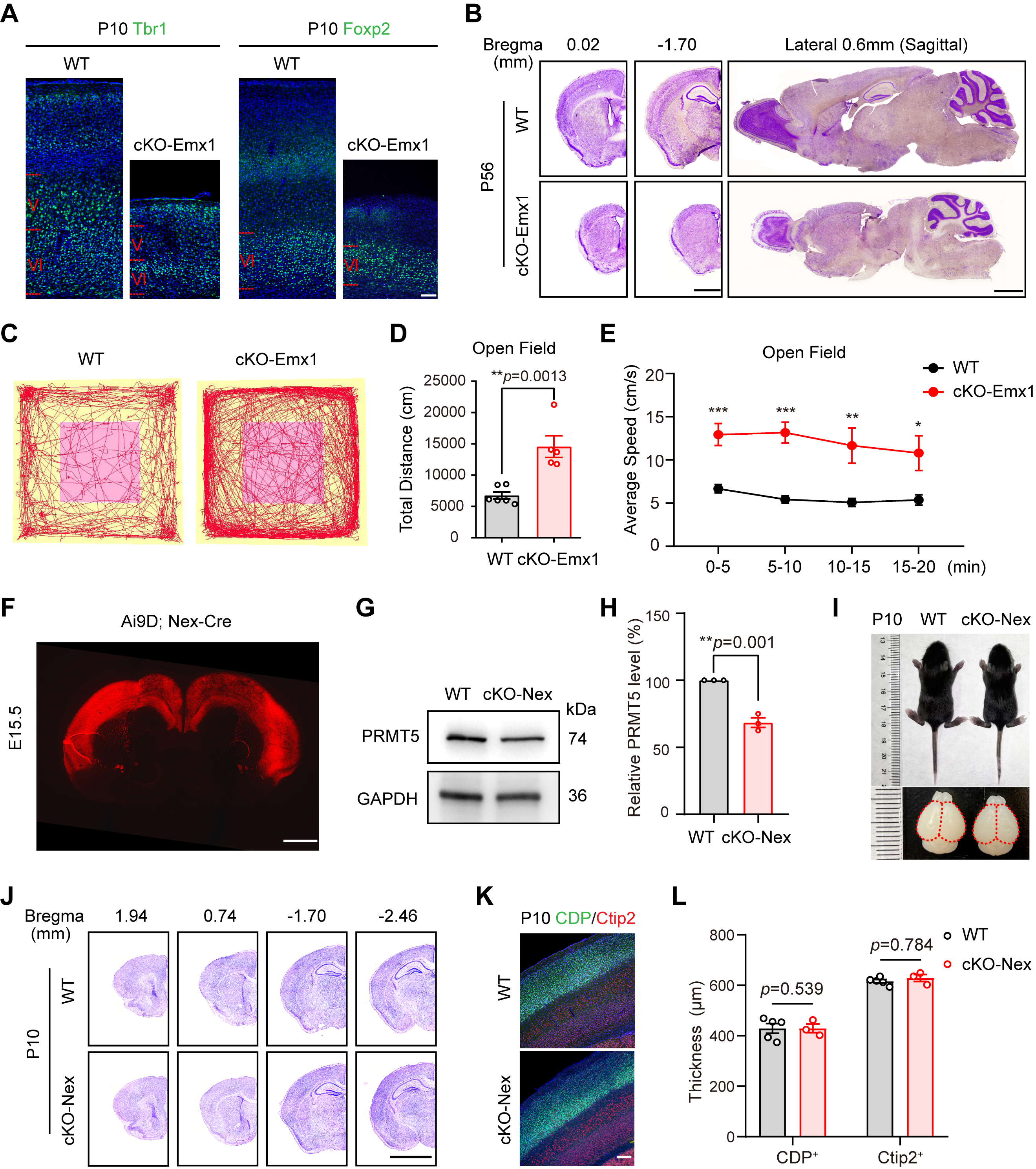
**

**Fig. S1** Motor dysfunction in cKO-Emx1 mice and no visible morphological deficiency in cKO-Nex mice. **A** Representative images of Tbr1/Foxp2 staining of WT and cKO-Emx1 brain slices. Scale bar, 100 μm. **B** Nissl staining of P56 WT and cKO-Emx1 brain slices. Scale bar, 1 mm. **C** Tracing of WT and cKO-Emx1 mouse locomotion in open field experiments (P56, males; WT: 6 mice, cKO-Emx1: 5 mice). **D** Statistical results of total movement distance. **E** Statistical results of the average speed of mice moving in the open field. **F** Representative image of E15.5 *Ai9D; Nex-Cre* brain sections. **G, H** Western blotting and quantification of PRMT5 expression in cKO-Nex and WT cortices (P10, males; N = 3 mice per genotype; data from 3 independent experiments). PRMT5 levels were normalized to GAPDH. The expression level in WT mice was set to 100%. Two-tailed unpaired *t* test. **I** Representative body and brain images of WT and cKO-Nex mice at P10. **J** Nissl staining of WT and cKO-Nex brain slices (P10, males; N = 3 mice per genotype). Scale bar, 1 mm. **K** Representative image of CDP/Ctip2 and Foxp2 staining of WT and cKO-Nex brain slices. Scale bar, 200 μm. **L** Quantification of CDP^+^ layer and Ctip2^+^ layer thickness. One-way ANOVA with Tukey’s post hoc test. For quantification, at least 3 animals for each genotype were analyzed. Mean ± SEM; **p* < 0.05; ***p* < 0.01; ****p* < 0.001.

**
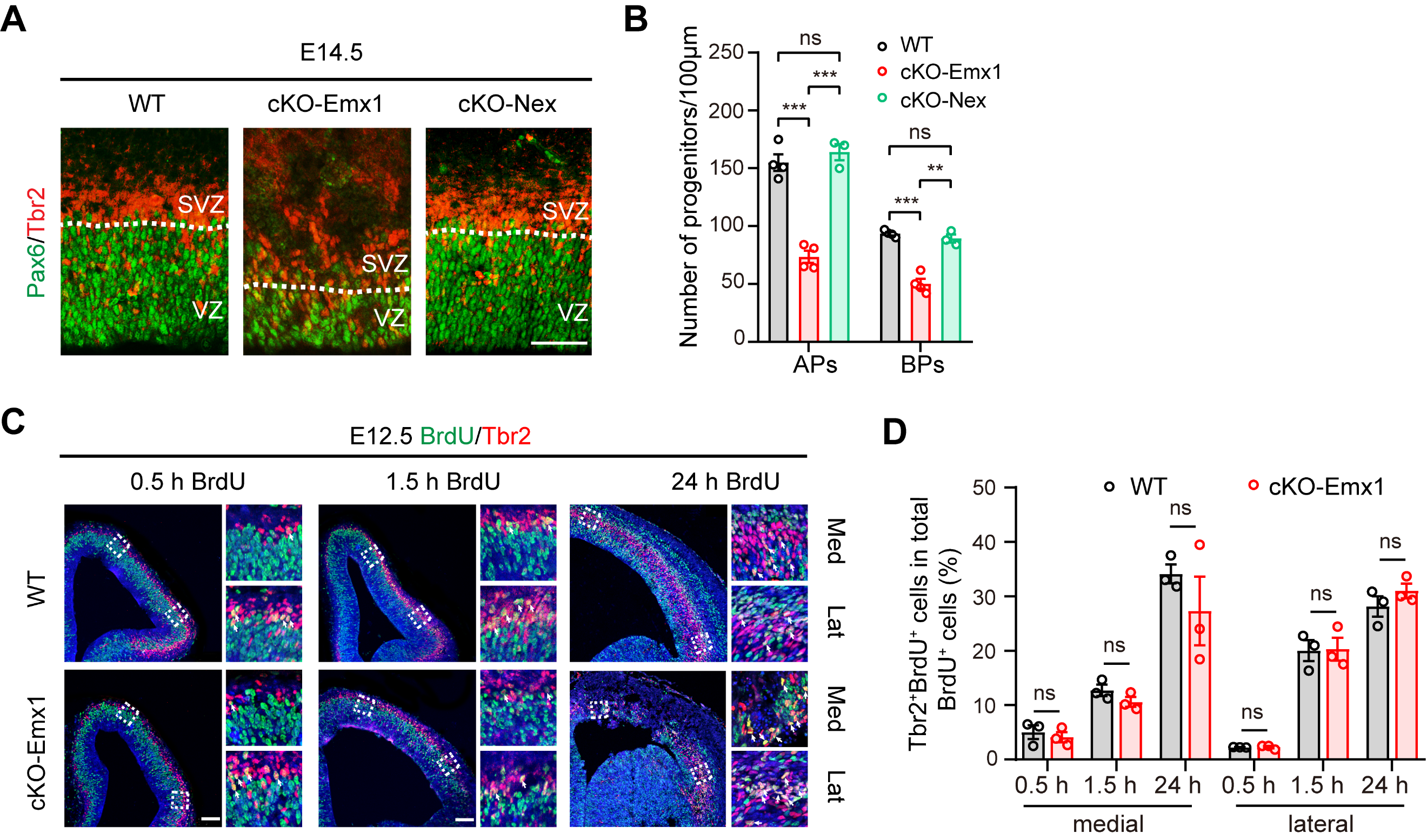
**

**Fig. S2** PRMT5 deletion in neurons showed no abnormal phenotype. **A** Representative images of Pax6/Tbr2 staining of E14.5 WT, cKO-Emx1 and cKO-Nex brains. The boundary of the SVZ and VZ is outlined by the white dashed line. Scale bar, 50 μm. **B** Quantification results of Pax6^+^ APs and Tbr2^+^ BPs within a radial column of 100 μm width. One-way ANOVA with Tukey’s post hoc test. **C** Brain sections were stained with BrdU (green) and Tbr2 (red) antibodies. Scale bar, 100 μm. Images on the right panel are enlarged views in the dashed boxes. **D** Percentage of BrdU^+^Tbr2^+^ cells among total BrdU^+^ cells in WT and cKO-Emx1 cortices. One-way ANOVA with Tukey’s post hoc test.

*
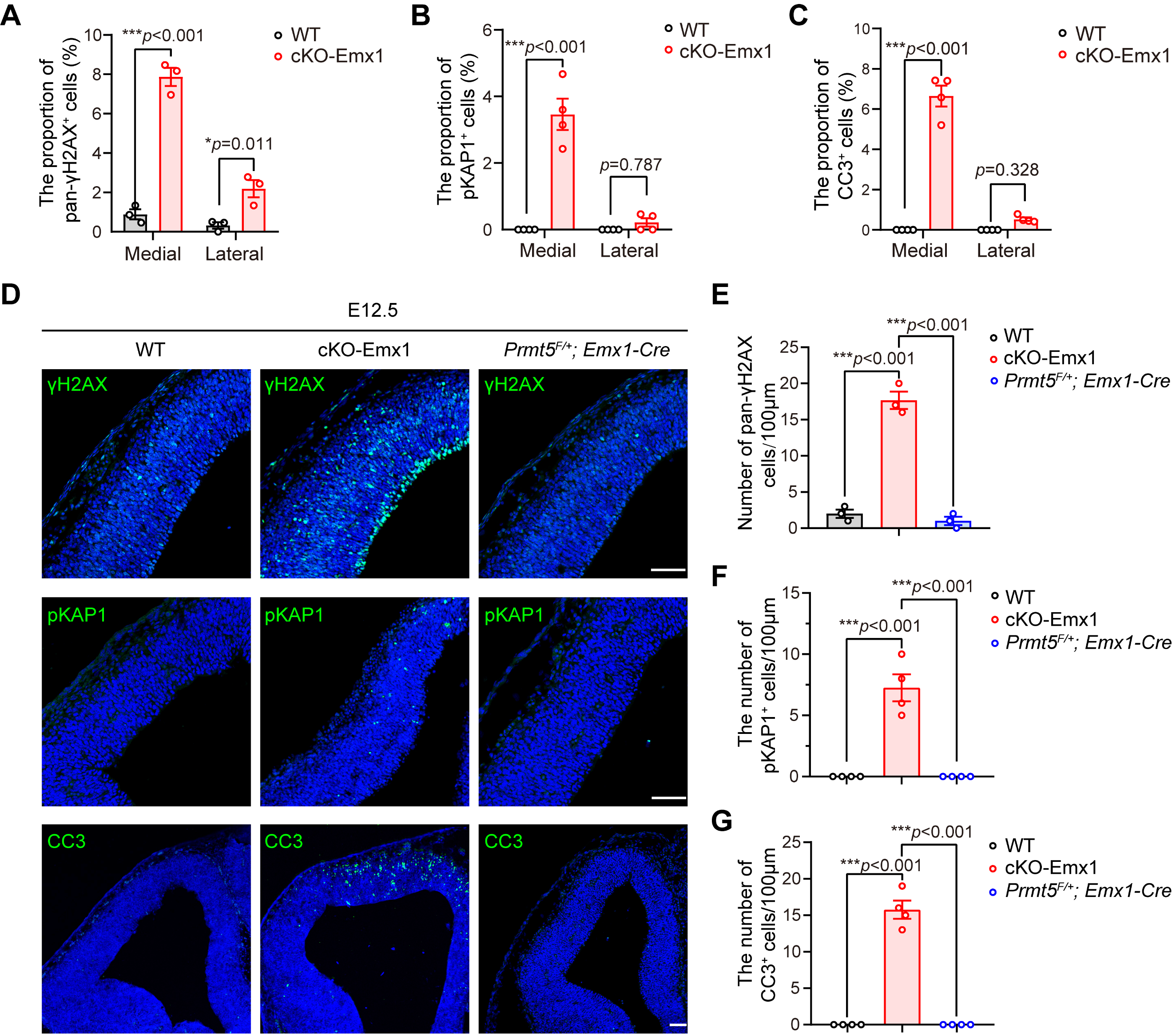
*

**Fig. S3** *Prmt5^F/+^*; *Emx1-Cre* mice showed no DSBs accumulation and apoptosis in cortex. **A-C** Quantification results of proportion of pan-γH2AX, pKAP1^+^ and CC3^+^ cells in E12.5 WT and cKO-Emx1 cortex from (**Fig. 3B**, **D** and **G**), respectively. **D** E12.5 WT, cKO-Emx1 and *Prmt5^F/+^*; *Emx1-Cre* brain sections were immunostained with γH2AX, pKAP1 and CC3, respectively. Scale bar, 100μm. **E,** **F,** **G** Quantification results of the number of pan-γH2AX, pKAP1^+^ and CC3^+^ cells within a column of 100 μm width of the E12.5 cortex from (**E**), respectively. One-way ANOVA with Tukey’s post hoc test. For quantification, at least 3 brains for each genotype were analyzed. Mean ± SD; **p* < 0.05, ***p* < 0.01, ****p* < 0.001.


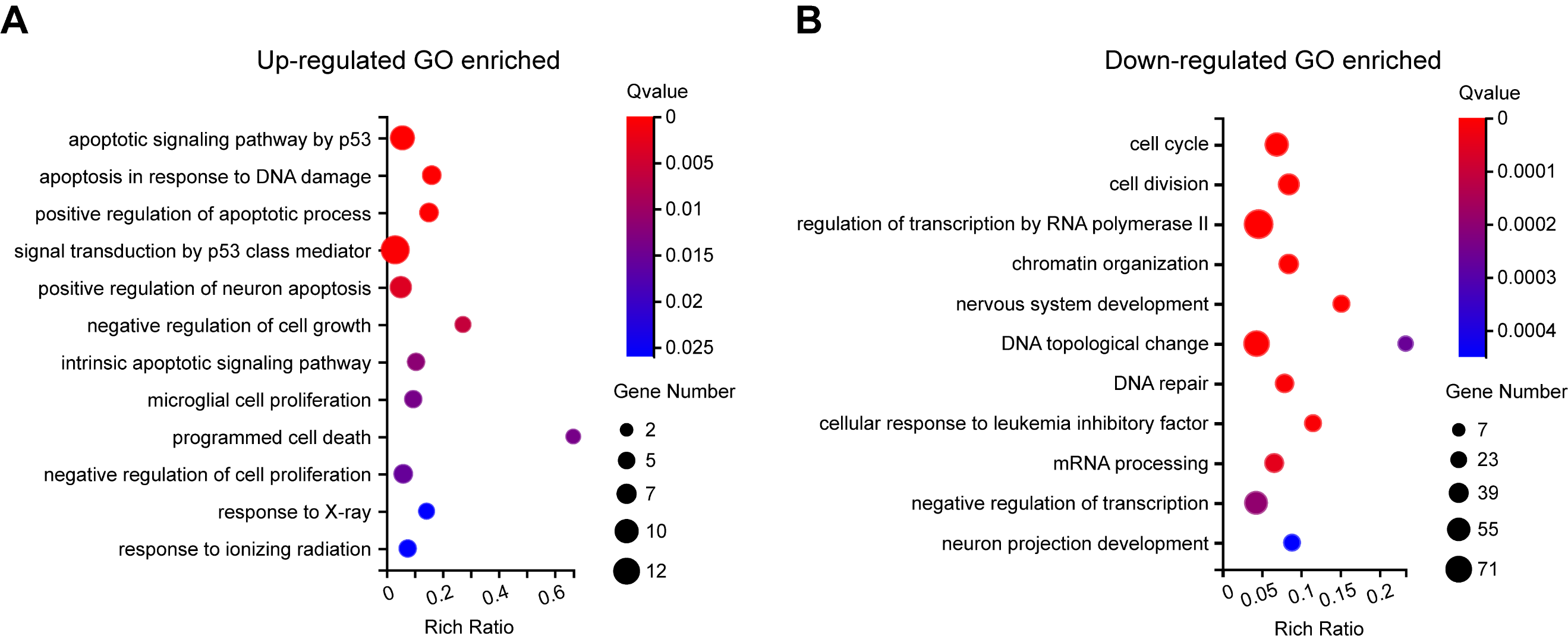


**Fig. S4** RNA seq analysis shows an abnormal transcriptomic signature in *Prmt5* cKO-Emx1 mice. **A** GO enrichment analysis for upregulated genes from E12.5 cortices RNA-seq datasets in *Prmt5* cKO-Emx1 mice. Upregulated genes in cKO-Emx1 are mainly enriched in “apoptotic signaling pathway by p53” and “apoptosis in response to DNA damage”. **B** GO enrichment analysis for downregulated genes from E12.5 cortices RNA-seq datasets in *Prmt5* cKO-Emx1 mice. Downregulated genes in cKO-Emx1 are mainly enriched in “cell cycle” and “DNA repair”.


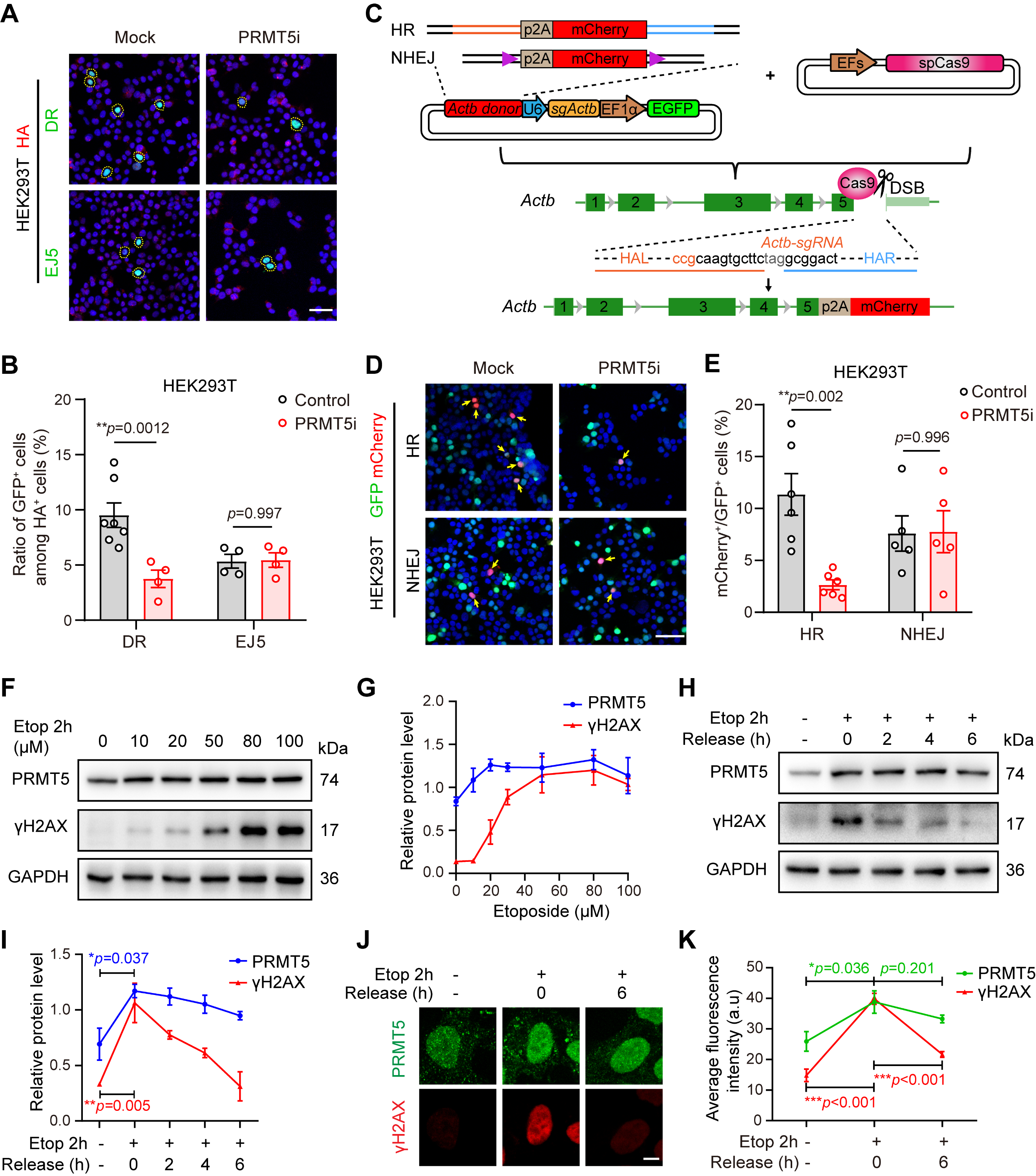


**Fig. S5** PRMT5 responds to DSBs and participates in DNA repair. **A** Representative fluorescence staining images of DMSO (Mock) or PRMT5 inhibitor (PRM5i) treated HEK293T cells after transfection for 72 h. Scale bar, 40 μm. **B** Quantification of the percentage of GFP^+^ cells among total transfected HA^+^ cells from (**A**). Two-tailed unpaired *t* test. **C** Schematic illustration of *in vitro* DSBs repair assay using CRISPR-Cas9. A genomic DSBs is induced by CRISPR-Cas9 within the coding region of *Actb* near the C terminus. Two reporter repair templates were designed such that repair of the DSBs by HR or NHEJ would lead to expression of mCherry. NSCs were transfected into CRISPR-Cas9 and reporter repair constructs, and were analyzed after 72 h. HAL/HAR, left/right homology arm. Two-tailed unpaired *t* test. For quantification, at least 3 independent experiments were analyzed. **D** Representative fluorescence staining images of DMSO (Mock) or PRMT5 inhibitor (PRM5i) treated HEK293T cells after transfection for 72 h. Scale bar, 20 μm. **E** Quantification of the percentage of mCherry^+^GFP^+^ cells among all the GFP^+^ cells from (**D**). Two-tailed unpaired *t* test. **F, G** Western blotting and quantification analyses of PRMT5 and γH2AX levels after 2 h of treatment with etoposide in cultured WT NSCs. Expression levels were normalized to GAPDH. One-way ANOVA with Tukey’s post hoc test. **H, I** Western blotting and quantification analyses of PRMT5 and γH2AX expression levels after etoposide removal in cultured NSCs. Expression levels were normalized to GAPDH. One-way ANOVA with Tukey’s post hoc test. **J** Representative immunofluorescence staining images of PRMT5 (green) and γH2AX (red) at the indicated time points in cultured WT NSCs. Scale bar, 5 μm. **K** Quantification of relative PRMT5 and γH2AX signal intensity in images from (**J**). One-way ANOVA with Tukey’s post hoc test. For quantification, data were from at least 3 independent experiments. Mean ± SEM; **p* < 0.05, ***p* < 0.01, ****p* < 0.001.

**
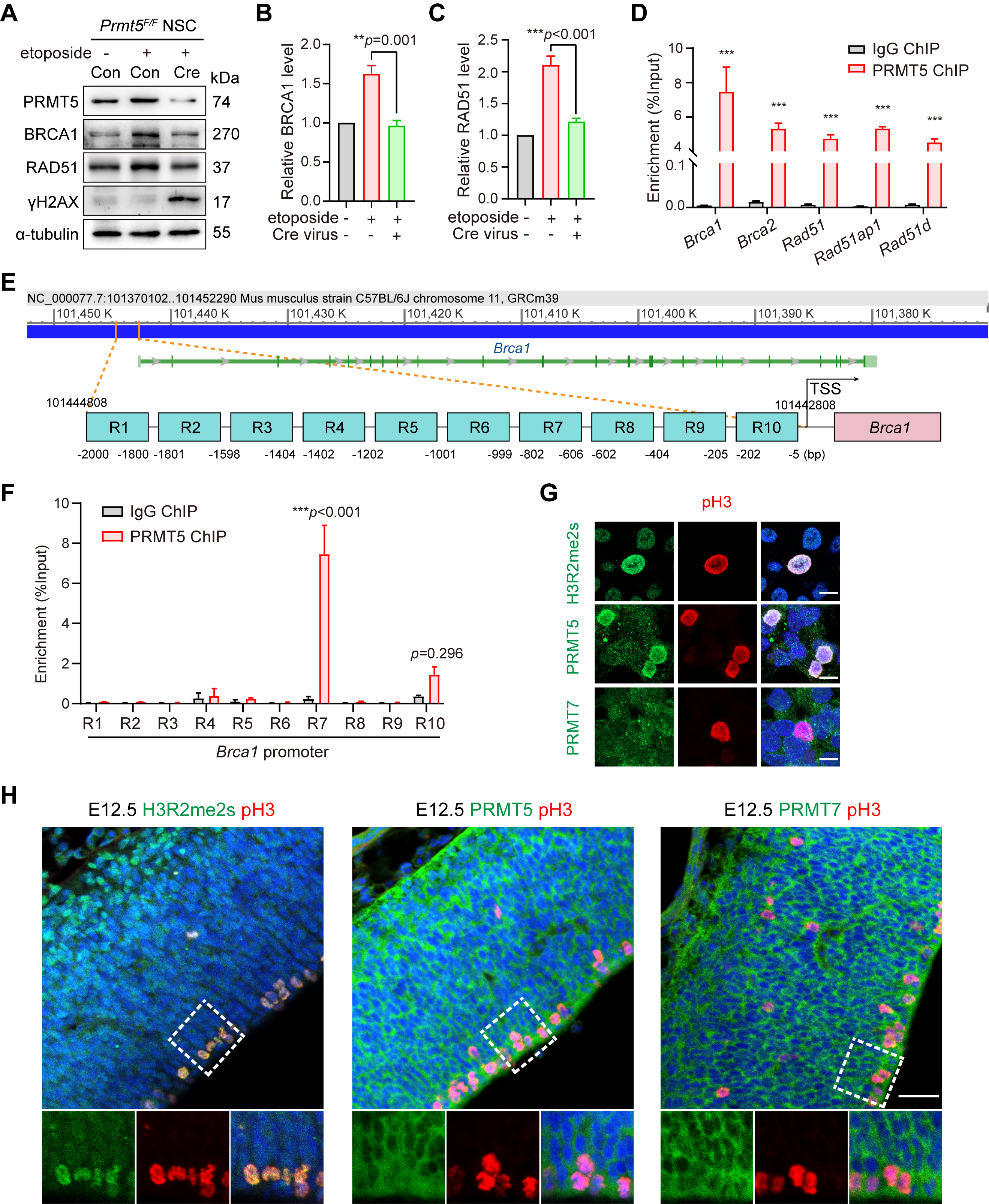
**

**Fig. S6** PRMT5 regulated DNA repair process via regulating the expression of BRCA1. **A** Western blotting showed that PRMT5 deletion abolished BRCA1 overexpression in response to etoposide treatment. **B, C** Quantification of BRCA1 and RAD51 levels via Western blotting from (**A**). Expression levels were normalized to α-tubulin. One-way ANOVA with Tukey's post hoc test. **D** Quantification of ChIP-qPCR results showed PRMT5 binds to the promoters of HR-related genes as indicated. Two-tailed unpaired t test. **E** Schematic of the promoter region of *Brca1* in the ChIP-qPCR experiment. The amplified regions (R) and their positions are labeled. The transcription start site (TSS) is shown with an arrow. **F** Quantification of ChIP-qPCR results between each *Brca1* promoter region and PRMT5 in NSCs. For each biological replicate, the value for IP was normalized to the value for IgG to calculate the fold change. Two-tailed unpaired *t* test. **G** Representative immunofluorescence staining images of H3R2me2s, PRMT5, PRMT7(green) and pH3 (red) in HEK293T cells, respectively. Scale bar, 5 μm. **H** E12.5 WT brain slices were immunostained with H3R2me2s, PRMT5, PRMT7(green) and pH3 (red), respectively. Images on the lower panel are enlarged views in the dashed boxes. Scale bar, 50 μm. Mean ± SEM; ***p* < 0.01, ****p* < 0.001.


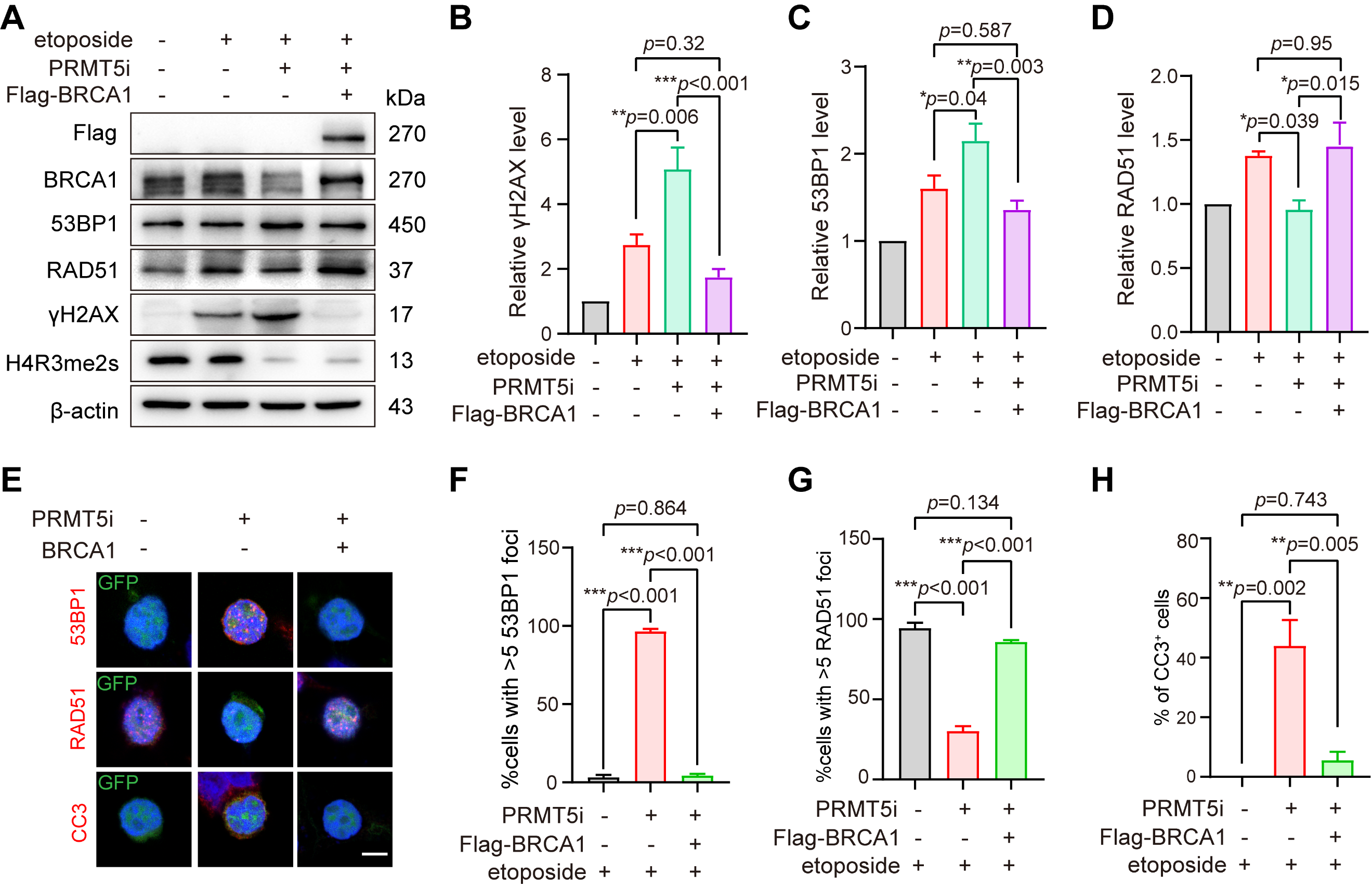


**Fig. S7** BRCA1 overexpression rescues the disrupted HR repair in PRMT5 deficient NSCs. **A** Western blotting analysis of 53BP1, RAD51 and γH2AX expression levels after etoposide and PRMT5 inhibition in cultured NSCs. **B-D** Quantification of γH2AX (**B**), 53BP1 (**C**) and RAD51 (**D**) levels via Western blotting from (**A**), respectively. Expression levels were normalized to β-actin. One-way ANOVA with Tukey’s post hoc test. **E** Representative images of 53BP1 (red, upper), Rad51 (red, medial) and CC3 (green, lower) immunostaining results. Scale bar, 4 μm. **F** Quantification of the percentage of cells with >5 53BP1 foci from (**E**). One-way ANOVA with Tukey’s post hoc test. **G** Quantification of the percentage of cells with >5 Rad51 foci from (**E**). One-way ANOVA with Tukey’s post hoc test. **H** Quantification of the percentage of CC3^+^ cells from (**E**). One-way ANOVA with Tukey’s post hoc test. Mean ± SEM; **p* < 0.05, ***p* < 0.01, ****p* < 0.001.

**Supplemental table 1. The list of primers for q-PCR.**

| **Primer name** | **Primer sequence** |
| --- | --- |
| *Gapdh*-F | 5’-AGGTCGGTGTGAACGGATTTG-3’ |
| *Gapdh*-R | 5’-TGTAGACCATGTAGTTGAGGTCA-3’ |
| *Prmt5*-F | 5’-CTGAATTGCGTCCCCGAAATA-3’ |
| *Prmt5*-R | 5’-AGGTTCCTGAATGAACTCCCT-3’ |
| *Rad51*-F | 5’-AAGTTTTGGTCCACAGCCTATTT-3’ |
| *Rad51*-R | 5’-CGGTGCATAAGCAACAGCC-3’ |
| *Rpa1*-F | 5’-ACATCCGTCCCATTTCTACAGG-3’ |
| *Rpa1*-R | 5’-CTCCCTCGACCAGGGTGTT-3’ |
| *Brca1*-F | 5’-CTGCCGTCCAAATTCAAGAAGT-3’ |
| *Brca1*-R | 5’-CTTGTGCTTCCCTGTAGGCT-3’ |
| *Brca2*-F | 5’-ATGCCCGTTGAATACAAAAGGA-3’ |
| *Brca2*-R | 5’-ACCGTGGGGCTTATACTCAGA-3’ |
| *Atm*-F | 5’-GATCTGCTCATTTGCTGCCG-3’ |
| *Atm*-R | 5’-GTGTGGTGGCTGATACATTTGAT-3’ |
| *Lig4*-F | 5’-ATGGCTTCCTCACAAACTTCAC-3’ |
| *Lig4*-R | 5’-TTTCTGCACGGTCTTTACCTTT-3’ |
| *Xrcc4*-F | 5’-CTGGAGGAGAGTACCAAACCT-3’ |
| *Xrcc4*-R | 5’-CTGGGGTAGTGAAGAGGCAAG-3’ |
| *Nhej1*-F | 5’-TGGGCATGGTTACAACTTGC-3’ |
| *Nhej1*-R | 5’-AACCGTGCTTGGTGATAGACA-3’ |
| *Trp53-F* | 5’-GTCACAGCACATGACGGAGG-3’ |
| *Trp53-R* | 5’-TCTTCCAGATGCTCGGGATAC-3’ |
| *Cdkn1a-F* | 5’-CCTGGTGATGTCCGACCTG-3’ |
| *Cdkn1a-R* | 5’-CCATGAGCGCATCGCAATC-3’ |
| *Bax*-F | 5’-TGAAGACAGGGGCCTTTTTG-3’ |
| *Bax-R* | 5’-AATTCGCCGGAGACACTCG-3’ |
| *Box*-F | 5’-AGGTAGTGTCCCTGTATTCCG-3’ |
| *Box-R* | 5’-AAGGTCTTGCGTACAAACTCC-3’ |
| *Ddit4*-F | 5’-CAAGGCAAGAGCTGCCATAG-3’ |
| *Ddit4-R* | 5’-CCGGTACTTAGCGTCAGGG-3’ |
| *Zmat3*-F | 5’-TTCCTTTACCTAATCGGCCTTCA-3’ |
| *Zmat3-R* | 5’-TTCCTGCCCAAAAGCCTTCTG-3’ |

**Supplemental table 1 (continued). The list of primers for q-PCR.**

| **Primer name** | **Primer sequence** |
| --- | --- |
| *Cdkn1a-F* | 5’-CCTGGTGATGTCCGACCTG-3’ |
| *Cdkn1a-R* | 5’-CCATGAGCGCATCGCAATC-3’ |
| *Bax*-F | 5’-TGAAGACAGGGGCCTTTTTG-3’ |
| *Bax-R* | 5’-AATTCGCCGGAGACACTCG-3’ |
| *Box*-F | 5’-AGGTAGTGTCCCTGTATTCCG-3’ |
| *Box-R* | 5’-AAGGTCTTGCGTACAAACTCC-3’ |
| *Ddit4*-F | 5’-CAAGGCAAGAGCTGCCATAG-3’ |
| *Ddit4-R* | 5’-CCGGTACTTAGCGTCAGGG-3’ |
| *Zmat3*-F | 5’-TTCCTTTACCTAATCGGCCTTCA-3’ |
| *Zmat3-R* | 5’-TTCCTGCCCAAAAGCCTTCTG-3’ |
| *Tbr2*-F | 5’-GCGCATGTTTCCTTTCTTGAG-3’ |
| *Tbr2*-R | 5’-GGTCGGCCAGAACCACTTC-3’ |
| *Pax6*-F | 5’-TACCAGTGTCTACCAGCCAAT-3’ |
| *Pax6*-R | 5’-TGCACGAGTATGAGGAGGTCT-3’ |
| *DCX*-F | 5’-CATTTTGACGAACGAGACAAAGC-3’ |
| *DCX*-R | 5’-TGGAAGTCCATTCATCCGTGA-3’ |
| *Neurod1*-F | 5’-ATGACCAAATCATACAGCGAGAG-3’ |
| *Neurod1*-R | 5’-TCTGCCTCGTGTTCCTCGT-3’ |

**Supplementary table 2. The list of primers for ChIP q-PCR.**

| **Primer name** | **Primer sequence (5'-3')** |
| --- | --- |
| *Brca1*-F | GATGCAATAAGCCGCAACTGG |
| *Brca1*-R | CCTCTTCCGTCTCTTTCCTTTTACG |
| *Brca2*-F | GAGAAGAGAACACACACTCCAGC |
| *Brca2*-R | GGTATTTCTCAGTGTGGCGAAAG |
| *Rad51*-F | GGATCCGCGAACGTAGATTCG |
| *Rad51*-R | GTCAGCTTTTGGCACTTCTGGTC |
| *Rad51ap1*-F | GGATCCGCGAACGTAGATTCG |
| *Rad51ap1*-R | GTTGGGACATAGGGGCACTTG |
| *Rad51d*-F | GCTTTGCTGCTTCTTGACACC |
| *Rad51d*-R | CCAGGGAGCTTACTGTGAGC |
| m*Brca1*-R1-F: 1 | AACCAGAAAAACAAAGTGACCA |
| m*Brca1*-R1-R: 201 | ACTAGAACCGCCTACTTCCT |
| m*Brca1*-R2-F: 202 | TTAGCTGGCCAATATACAGGT |
| m*Brca1*-R2-R: 405 | ACGTGAGTCAATAGGTATGAG |
| m*Brca1*-R3-F: 403 | CGTTCATGTGGCTAAGTCAG |
| m*Brca1*-R3-R: 599 | TCCAAATTAGAAAGGCATCGTT |
| m*Brca1*-R4-F:601 | GGTCCTAGCTCAAGATATCTT |
| m*Brca1*-R4-R:801 | GAAAAGGTCTCGATCCCCC |
| m*Brca1*-R5-F:801 | CTTAGCAGAAACTGGCCCTT |
| m*Brca1*-R5-R: 1002 | GAAGTAGGGGCACGCCGT |
| m*Brca1*-R6-F: 1004 | AGGTAACAGTGGTAGCTTCC |
| m*Brca1*-R6-R: 1199 | TGAGGGGCAGATAGAAAGAG |
| m*Brca1*-R7-F: 1201 | AATAACCTCTCTACCGATTGG |
| m*Brca1*-R7-R: 1397 | GTAACTAGTCCGCCAATGTC |
| m*Brca1*-R8-F: 1401 | AGAATCGCGGGGCTGGAC |
| m*Brca1*-R8-R: 1599 | GGGAAAAGAGCTTCTCACAC |
| m*Brca1*-R9-F: 1599 | CGTAAGACTACACTTTCCAAG |
| m*Brca1*-R10-F: 1801 | CGATTCCCACCCTTTGTACA |
| m*Brca1*-R10-R: 1998 | AGGGAGGAGCGAGTTGGG |
